# Comparative Life Cycle Assessment for the Fabrication of Polysulfone Membranes Using Slot Die Coating as a Scalable Fabrication Technique

**DOI:** 10.3390/polym17172363

**Published:** 2025-08-30

**Authors:** David Lu, Isaac Oluk, Minwoo Jung, Sophia Tseng, Diana M. Byrne, Tequila A. L. Harris, Isabel C. Escobar

**Affiliations:** 1 Department of Chemical and Materials Engineering, University of Kentucky, Lexington, KY 40506, USA; david.lu@uky.edu (D.L.); sts224@uky.edu (S.T.); 2 Department of Civil Engineering, University of Kentucky, Lexington, KY 40506, USA; isaacoluk@uky.edu (I.O.); dianabyrne@uky.edu (D.M.B.); 3 George W. Woodruff School of Mechanical Engineering, Georgia Institute of Technology, Atlanta, GA 30332, USA; minpal3@gatech.edu (M.J.); tharris3@gatech.edu (T.A.L.H.)

**Keywords:** membranes, eco-friendly solvents, roll-to-roll, sustainability, life cycle assessment

## Abstract

Despite the emergence of eco-friendly solvents and scalable methods for polymeric membrane fabrication, studies on the impacts of solvent synthesis and manufacturing scale-up have not been conducted. To this end, a life cycle assessment (LCA) was developed with the goal of determining the global environmental and health impacts of producing polysulfone (PSf) membranes with the solvents PolarClean and γ-valerolactone (GVL) via doctor blade extrusion (DBE) and slot die coating (SDC). Along with PolarClean and GVL, dimethylacetamide (DMAc) and N-methyl-2-pyyrolidone (NMP) were included in the LCA as conventional solvents for comparison. The dope solution viscosity had a major influence on the material inventories; to produce a normalized membrane unit on a surface area basis, a larger quantity of PSf-PolarClean-GVL materials was required due to its high viscosity. The life cycle impact assessment found electricity and PolarClean to be major contributing parameters to multiple impact categories during membrane fabrication. The commercial synthesis route of PolarClean selected in this study required hazardous materials derived from petrochemicals, which increased its impact on membrane fabrication. Due to more materials being required to fabricate membranes via SDC to account for tool fluid priming, the PSf-PolarClean-GVL membrane fabricated via SDC exhibited the highest impacts. The amount of electricity and concentration of PolarClean were the most sensitive parameters according to Spearman’s rank coefficient analysis. A scenario analysis in which the regional energy grid was substituted found that using the Swedish grid, which comprises far more renewable technologies than the global and US energy grids, significantly lowered impacts in most categories. Despite the reported eco-friendly benefits of using PolarClean and GVL as alternatives to conventional organic solvents, the results in this study provide a wider perspective of membrane fabrication process impacts, highlighting that upstream impacts can counterbalance the beneficial properties of alternative materials.

## 1. Introduction

Organic solvents commonly used in commercial polysulfone membrane fabrication via nonsolvent-induced phase separation are becoming increasingly restricted due to their reported toxic, carcinogenic, irritant, and other operating hazard properties [1]. These solvents, including dimethylacetamide (DMAc), dimethylformamide (DMF), and N-methyl-2-pyrrolidone (NMP) as commonly used examples, are also derived from fossil fuel sources that limit their sustainability [1]. In the European Union, DMAc, DMF, and NMP manufacturing and usage are now limited under Registration, Evaluation, Authorization, and Restriction of Chemicals (REACH) regulations, including NMP restrictions since 2018, DMF restrictions since 2023, and DMAc restrictions by 2026 [2,3,4]. In 2024, the US Environmental Protection Agency (EPA) announced a proposed rule that would implement similar restrictions on NMP manufacturing, processing, and distribution [5]. While no timeline for further action has been established, other EPA-led chemical restrictions have occurred within a 5-year timeline [2].

The growing regulations on commercially available solvents have motivated the emergence of membrane fabrication studies using non-hazardous and eco-friendly solvents. While a myriad of eco-friendly alternatives has been identified, the additional criterion of large-scale production is important to support the economic feasibility of substitution. Among this group of alternatives, methyl 5-(dimethylamino)-2-methyl-5-oxopentanoate (Rhodiasolv^®^ PolarClean) and γ-valerolactone (GVL), two nontoxic and biodegradable solvents, are considered suitable solvents for producing polysulfone (PSf) membranes. PolarClean is the chemical valorization product of methylglutaronitrile (MGN), a byproduct of nylon-6,6 synthesis [3]; GVL is produced from levulinic acid sourced from lignocellulosic biomass [4]. The use of these solvents individually results in PSf membranes hydrolyzing upon formation or experiencing pore collapse during filtration. However, their use in combination has been shown to produce durable PSf membranes with permeability and solute rejection similar to PSf-NMP membranes [3,5,6].

Beyond the lab-scale demonstration of PSf-PolarClean-GVL membrane fabrication, proving the scalability and sustainability of this process is imperative to determining commercial viability. While commonly utilized in lab-scale studies, polymeric membrane fabrication via doctor blade extrusion (DBE) is difficult to scale up and integrate with a roll-to-roll (R2R) system for continuous casting [7]. Slot die coating (SDC), a manufacturing method commonly used to produce thin films, has been shown to also produce defect-free polymeric membranes and is compatible with an R2R configuration, paving the way for the manufacturing scale-up of PSf membranes with novel materials [8]. Using SDC, studies by Dong et al. [7] and Lu et al. [8] have reported the fabrication of PSf-PolarClean-GVL membranes that possess membrane morphology, surface pore size, total porosity, water permeability, and solute rejection similar to membrane samples prepared via DBE [7,8,9].

Studies on polymeric membrane fabrication using eco-friendly solvents or scalable fabrication techniques have largely focused on investigating membrane properties and performance, though investigating the environmental and health impacts is another key element to evaluating commercial viability. While it may be difficult to definitively assess material and process sustainability, it is possible to perform a systematic analysis of the produced impacts. To this end, a life cycle assessment (LCA) is an environmental management tool for quantifying the environmental and health impacts over the life cycle of a product, process, or activity to inform decision making [10]. Depending on the assessment, the life cycle can include raw material extraction, manufacturing, distribution, operation, maintenance, and end-of-life stages [10]. With the development of a standardized methodological framework and terminology by the International Organization for Standardization (ISO 14040 and ISO 14044), the prominence and merit of LCAs have significantly grown [11,12]. As part of the ISO-issued framework, a standard LCA must include a goal and scope definition, inventory analysis, life cycle impact assessment, and interpretation [10]. Using this common methodology, LCAs have increasingly become a core element in product development and environmental policy worldwide, including applications in waste incineration, building materials, and military systems [13].

Since their standardization, LCAs have also become a tool for investigating the impacts of polymeric membrane fabrication with novel materials. In particular, recent LCAs on membrane fabrication have compared the impacts of membrane fabrication when substituting conventional solvents with eco-friendly alternatives, including ethylene carbonate (EC), ethyl acetate (EA), γ-butyrolactone (GBL), PolarClean, and GVL [14,15,16]. Yadav et al. [14] reported that conventional solvents heavily contribute to global warming potential, human toxicity, and fossil resource scarcity impacts during membrane fabrication and that substitution with EC significantly can reduce the solvent’s relative contribution to these impacts. Similarly, Hong et al. [15] found that substituting NMP with GBL or EA reduces membrane fabrication impacts by at least 10%, particularly during dope solution preparation. However, Fionah et al. [5,16] have reported that PSf membranes solely prepared with PolarClean exhibit structural deficiencies during water filtration, while those solely prepared with GVL hydrolyze following casting. Follow-up studies have reported the formation of more durable PSf membranes using a 3:1 ratio of PolarClean and GVL, though the changes in impacts when using both have not been studied [5,6,8].

Another limitation in the existing LCA literature is that LCAs in the field of membrane science have only focused on DBE as the fabrication technique. To date, existing LCAs on polymeric membrane fabrication are limited to bench-scale fabrication techniques. While some have calculated the impacts of large-scale membrane production by increasing the functional unit, assessments continue to use DBE as the fabrication technique despite its difficulty for scaled fabrication; in particular, it is difficult to meter without additional tooling when part of an R2R system [17,18]. Conversely, LCAs on SDC are limited to applications in solar cell manufacturing [19,20,21,22]. Zhao et al. [19] and Vesce et al. [22] found impacts to be lower by 1–3 magnitudes of order when substituting spin coating with SDC to fabricate perovskite solar cells, though it remains to be seen if applying SDC for polymeric membrane fabrication yields similar results. With the difference in material and machinery input when using SDC, new LCAs are needed to determine if such a scalable technique can still be used to fabricate PSf membranes without creating significantly higher impacts. Thus, the goal of this study is to build on the previous findings of Fionah et al. [16] by performing an LCA that estimates the global environmental and health impacts of producing PSf-PolarClean-GVL membranes using SDC and comparing them to conventional solvents and the bench-scale DBE technique. The assessment also pinpoints the parameters that have significant impacts and the need for further analysis. Ultimately, the life cycle impacts identified in this LCA can be used to formulate strategies to reduce impacts from the membrane fabrication scale-up process.

## 2. Experimental

### 2.1. Materials

Polysulfone (PSf, average Mw 35,000 by LS, Mn 16,000 by MO, pellets) and N,N-Dimethylacetamide (ReagentPlus^®^, ≥99%) were purchased from Sigma-Aldrich (St. Louis, MO, USA). Rhodiasolv^®^ PolarClean was provided by Solvay. γ-valerolactone (ReagentPlus^®^, 99%) was purchased from Sigma-Aldrich (Fair Lawn, NJ, USA). N-methyl-2-pyrrolidone (NMP) was purchased from VWR (Visalia, CA, USA). Tap water and DI water were used as the nonsolvent during membrane fabrication.

### 2.2. Membrane Fabrication

Dope solutions and membrane samples were prepared to determine the material inventory amounts. To produce an ultrafiltration (UF) flat sheet membrane, a dope solution containing 17 wt.% PSf and a 3:1 ratio of PolarClean/GVL was first prepared based on previous studies [5,6,7,8]. PSf was added to the solvents and mixed for 24 h at a temperature of 80 °C and spin rate of 200 rpm using a VWR 7X7 CER Hot/Stir 120 stir plate.

The dope solution was cast onto a glass plate using a doctor blade (Micrometer Adjustable Film Applicator 250 mm, MTI Corp, Richmond, CA, USA) and then immersed in a tap water nonsolvent bath. Thus, the membranes were formed via nonsolvent-induced phase separation (NIPS), in which the solvent and nonsolvent mix and de-mix during the immersion step, forming a polymer-rich phase that becomes the PSf matrix and a polymer-poor phase that forms the membrane pores [1]. The resulting membrane sheet was immersed in DI water for storage.

The slot die system used to fabricate membranes for LCA analysis consisted of a single-layer slot die and an R2R system. The single-layer slot die was attached to the R2R system for high output and a continuous system for scalable membrane fabrication. The single-layer slot die consisted of two dies, upstream and downstream, with a shim in between to create a slot at a specific thickness that is directly related to the thickness of the shim. The slot width had a dimension of 4 inches and a thickness of 210 µm, since the shim thickness was 210 µm. The dope solution was then pumped with a syringe pump to deliver the dope solution into a slot die at a specific flow rate. When the dope solution was filled enough inside the slot die reservoir, the dope solution exited out at the slot, creating a meniscus, with the dope solution bridging the slot die lip and the substrate. The substrate was moved at a specific speed to create Couette flow at the meniscus to create an evenly distributed wet film when the dope solution was dragged beyond the slot die downstream lip. The coated wet film with dope solution was then phase inverted through NIPS using deionized water as a nonsolvent. The porous membrane was fabricated after about 20 min of phase inversion bath.

### 2.3. Life Cycle Assessment (LCA)

A life cycle assessment (LCA) was completed to estimate the environmental and human health impacts of producing PSf membranes using eco-friendly solvents and different membrane fabrication techniques. Impacts of membranes prepared with PolarClean and GVL (eco-friendly solvents) were compared with membranes prepared with DMAc and NMP, two conventional organic solvents commonly used in membrane production. To investigate the impacts from using a scalable fabrication technique, the LCA compared doctor blade extrusion (DBE) and slot die coating (SDC). The complete list of membrane samples analyzed in the LCA is listed in Table 1. While there are similarities to the membrane types compared in Fionah et al. [16], such as the PSf/NMP membrane, the 3:1 mixture of PolarClean-GVL in M3 and M6 is a unique configuration for this LCA, as previous membrane configurations solely used PolarClean or GVL. Inventory data for the LCA was sourced from lab-scale membrane fabrication experiments using both DBE and SDC. MATLAB R2024b was used to build a life cycle inventory, perform the life cycle impact assessment, and execute uncertainty and sensitivity analyses.

#### 2.3.1. Goal and Scope

**Figure 1:**
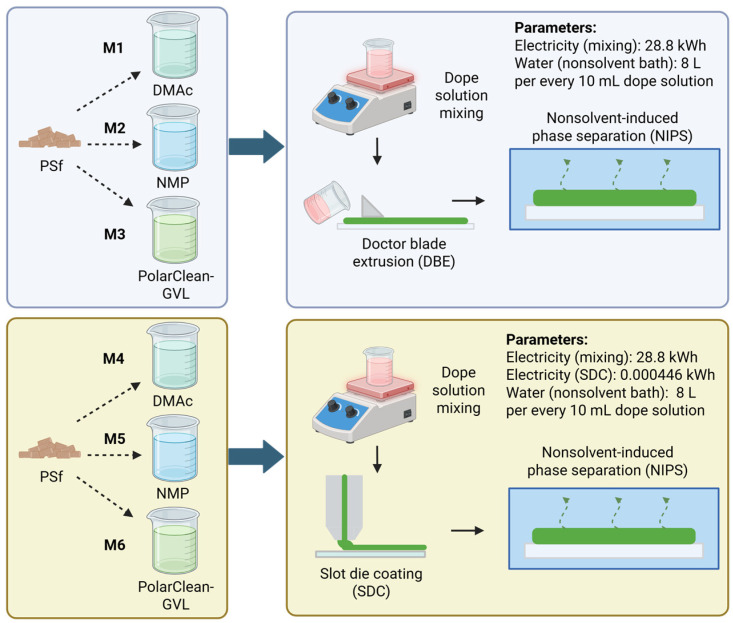
Overall framework of the LCA.

The goal of the LCA was to compare the global environmental and human health impacts of producing lab-scale flat sheet PSf membranes using different solvents and a conventional lab-scale versus a scalable fabrication method. The LCA system boundaries were “cradle-to-gate,” focusing on the impacts from input material synthesis to the preparation of the dope solution and subsequent membrane fabrication; thus, the impacts from membrane operation and end-of-life were not considered. The overall framework and boundaries are visualized in Figure 1. Impacts were normalized across all membranes by defining a functional unit of 1 m^2^ of viable (i.e., defect-free) flat sheet membrane being produced in order to be consistent with previous studies that applied LCA to polymeric membrane fabrication [23,24].

#### 2.3.2. Assumptions

Assumptions were established for the LCA about the extent of materials and manufacturing inputs and processes. Impacts from producing the membrane fabrication equipment and material transport to the lab were excluded from the assessment. Since the LCA was designed on a global production basis, material transportation between specific locations was discounted in the assessment. Each membrane included an electricity input of 28.8 kWh calculated from the duration of mixing the dope solution. Electricity input for heating the PSf-PolarClean-GVL dope solutions was excluded, as it was impractical to determine electricity input for heating as a function of temperature; the baseline power requirement of the stir plate was used in the LCA. Inventory values obtained from fabricating lab-scale membrane samples (i.e., approximately 0.08 m^2^ in area) were assumed to linearly scale to achieve the functional unit used in this study. During membrane fabrication, material loss due to solvent evaporation was excluded; given the short time duration between casting/coating and immersion, evaporative loss was considered negligible. Following experimental procedures from previous studies to produce these membranes, a volumetric ratio of 8 L of nonsolvent bath to 10 mL dope solution was set based on lab-scale membrane fabrication procedures [9,10]. The end boundary, defined as the membrane production (i.e., cradle-to-gate), was selected to more accurately compare with related LCAs. Other LCAs in this field have also used a cradle-to-grave system, as a multitude of operational applications would require additional assumptions and complicate the comparison of impacts. Based on water filtration demonstrations in previous studies that compared PSf-PolarClean-GVL membranes to PSf membranes with conventional solvents, we assumed a negligible difference in membrane performance between each type in this study [5,6,7,8].

#### 2.3.3. Life Cycle Inventory (LCI)

The general process for developing the life cycle inventory was adapted from Fionah et al. [16]. The LCI was developed using the Ecoinvent 3.5 inventory database within SimaPro v.9.0.0.49 PhD modeling software [25]. Ecoinvent was used to obtain background data from most materials used in membrane fabrication. All materials selected in Ecoinvent and the material amounts for each membrane are listed in Table S1 and Table S2, respectively. Breakdowns of production inputs for the parameters are listed in Table S3 through Table S8. Background data on the synthesis routes for PolarClean and GVL were obtained from the literature. The synthesis (Commercial Route A1) of PolarClean was sourced from Cseri and Szekely [26]. Following the approach of Fionah et al. [16], the production process for GVL (GVL Unit Process) was sourced from Han et al. [27] using kenaf fiber as cellulosic feedstock. Electricity input for the stir plate was obtained from the device specifications; the electricity input for the SDC equipment was measured using a MECHEER PM1 power energy meter (MECHEER, Shenzhen, China).

In both fabrication techniques, a measurable portion of the dope solution did not contribute to the formation of a viable membrane sheet. During DBE, residual dope solution is left on the doctor blade, and portions of the membrane sheet may contain surface defects (e.g., wrinkles and holes). During SDC, the first portion of the dope solution acts to prime the coater; as a result, the coating bead is initially unstable and results in surface defects. These “material losses” were included in the LCI to simulate a realistic membrane manufacturing process. To account for material losses during DBE, three PSf-DMAc, PSf-NMP, and PSf-PolarClean-GVL membrane samples were cast, and the weight of the doctor blade before and after casting was used to measure residual dope solution; the total surface area of the membrane samples containing defects was calculated to determine total dope solution quantity, and a factor was used to multiply the original LCI values to account for material loss. Images of the membrane samples and calculation results are presented in Figure S1 and Table S9, respectively.

#### 2.3.4. Life Cycle Impact Assessment (LCIA)

The life cycle impact assessment (LCIA) was conducted using the Tool for the Reduction and Assessment of Chemicals and Other Impacts (TRACI) 2.1 version 1.05 developed by the US Environmental Protection Agency and implemented using SimaPro version 9.0.0.49 [28]. All ten impact categories related to environmental and human health impacts were selected in TRACI: global warming potential (kg CO_2_ eq), ozone depletion (kg CFC-11 eq), smog (kg O_3_ eq), acidification (kg SO_2_ eq), eutrophication (kg N eq), ecotoxicity (CTUe), fossil fuel depletion (MJ surplus), carcinogenic toxicity (CTUh), noncarcinogenic toxicity (CTUh), and respiratory effects (kg PM_2.5_ eq). MJ surplus is defined as the amount of additional energy needed to extract one unit of a resource in the future [29]. CTUh denotes comparative toxic units for human toxicity potential and is defined as the estimated increase in morbidity in the total human population per unit mass of an emitted chemical [30]. CTUe denotes comparative toxic units for ecotoxicity potential and is defined as the estimated potentially affected fraction of species per unit mass of a chemical emitted integrated over time and volume [30]. PM_2.5_ denotes fine particulate matter with an aerodynamic diameter of 2.5 μm or less [31].

#### 2.3.5. Uncertainty and Sensitivity Analyses

The uncertainty analysis was completed by performing 10,000 Monte Carlo simulations programmed into MATLAB version R2020a in which uniform distributions with minimum and maximum values representing −30% and +30% of baseline values were created and used to produce impact distributions. The sensitivity analysis was completed to assess the relationships between the input materials and the resulting impact magnitudes using Spearman’s rank correlation coefficient. Spearman’s rank correlation coefficient values (ρ) measure the strength of a monotonic relationship between 2 sets of ranked data, ranging between 1.0 (a perfect correlation) and −1.0 (a perfect negative correlation) [1]. In an LCA, determining the ρ provides a quantitative measure for the variance in impact results caused by the assumption of variation in each input parameter [2]. The equation for determining ρ is found in Equation S1 in the SI.

#### 2.3.6. Scenario Analysis

In the scenario analysis, the electricity market was varied to determine if differences in the energy grid mix (e.g., solar, nuclear, wind, and fossil fuels) altered the impact results. In the materials inventory, the Ecoinvent process for electricity, medium voltage, was initially set to the global electricity market (GLO) and was substituted with the United States (US) and Swedish (SE) electricity markets. The Swedish electricity market was selected based on its notable differences in renewable energy technologies, including significantly greater portions of the energy mix being sourced from nuclear, wind, and hydropower compared to the global and US markets [32]. Additionally, the Swedish market was used in a scenario analysis reported by Yadav et al. [14] in a related study for comparison.

## 3. Results and Discussion

### 3.1. Influence of Membrane Fabrication on the Life Cycle Inventory

The dope solution dynamic viscosity measurements were collected using a rheometer (Rheometer DHR3, TA instruments, New Castle, DE, USA) at ambient temperature and over a shear range of 0–1000 s^−1^, which was based on procedures from a previous study [6]. In both fabrication techniques, the dope solution viscosity significantly influenced the size of the membrane sheets produced. At a constant shear rate of 27 s^−1^, dynamic viscosities of 17 wt.% PSf-DMAc, PSf-NMP, and PSf-PolarClean-GVL dope solutions were found to measure 0.069 Pa·s, 0.495 Pa·s, and 3.147 Pa·s, respectively. The viscosity results were consistent with those previously reported over shear rates of 1–10 s^−1^_,_ indicating enhanced polymer swelling as PSf mixed with eco-friendly solvents across a range of shear rates [5,6].

During DBE, a uniform shear force was applied to spread the cast solution across the glass plate to form the membrane. When casting the PSf-DMAc and PSf-NMP dope solutions, the low viscosity allowed the solutions to be spread further across the plate and produce larger membranes. In contrast, the membrane samples produced using the PSf-PolarClean-GVL dope solution were smaller due to the viscosity creating greater resistance during casting. The variation in membrane thickness was evident with samples ranging in thickness between approximately 0.8 and 1.0 mm, in which the solutions with PolarClean and GVL were thinner than those with DMAc and NMP. Thus, a larger material quantity was required to produce the functional unit for the PSf-PolarClean-GVL membranes. For this study, the spreading of the dope solution played a major role in determining the material inventory, since the functional unit was set on a surface area basis; while consistent with other LCAs on membrane fabrication, this basis does not take the membrane thickness into account, which would also influence the material inventory.

Additionally, the solution viscosity influenced the quantity of surface defects that formed on the membranes and residual solution left on the doctor blade. Based on casting experiments pictured in Figure S1 and summarized in Table S9, the dope solution viscosity was inversely related to the defective area, total defect dope solution volume, residual solution volume, and the material loss factor: 1.517, 1.133, and 1.0779 for M1, M2, and M3, respectively. This trend was consistent with reported literature on membrane fabrication, in which wrinkles were evidence of membrane shrinkage due to the solvent-nonsolvent de-mixing; as viscous solutions exhibit delayed de-mixing, shrinkage and wrinkle formation were minimized [33]. Ismail et al. [34] and Chung et al. [35] also reported that high dope solution viscosity was due to extensive polymer chain entanglement and essential to producing membranes with minimum defects. Thus, multiplying each material amount with the corresponding factor provided a more realistic material inventory for membrane fabrication by accounting for the formation of both viable and defective regions, with the latter ultimately being removed from the sheet. Despite M1 and M2 exhibiting greater areas of defects, the dope solution for M3 still required the largest solution amount and indicated that the total amount of required solution for M3 was greater than the required amount to compensate for defective areas of M1 and M2. As listed in Table S10, M1, M2, and M3 required dope solution amounts of 0.131 kg, 0.152 kg, and 0.307 kg, respectively.

A similar trend was found when developing the material inventories for membranes produced via SDC. As the coating bead must first reach a steady state to form a viable membrane, an initial portion of the solution was allocated for tool fluid loss, which was considered analogous to the solution volume that formed defective membrane areas and the residual dope solution volume during DBE. From lab-scale coating experiments, tool fluid loss amounts for M4, M5, and M6 were 9.4 g, 11.89 g, and 21.01 g, respectively. The relatively high viscosity of the PSf-PolarClean-GVL dope solution required increased pressure and solution to fill the SDC reservoir before exiting the slot, thereby increasing its tool fluid loss. When combined with the dope solution amounts for producing the membrane sheet and scaled to the functional unit, 0.913 kg, 0.979 kg, and 1.273 kg were required for M4, M5, and M6, respectively (Table S10). With both fabrication techniques, the larger dope solution amount due to viscosity resulted in larger material inventory amounts, potentially increasing the impacts.

### 3.2. Parameter Relative Contributions to Membrane Fabrication Impacts

**Figure 2:**
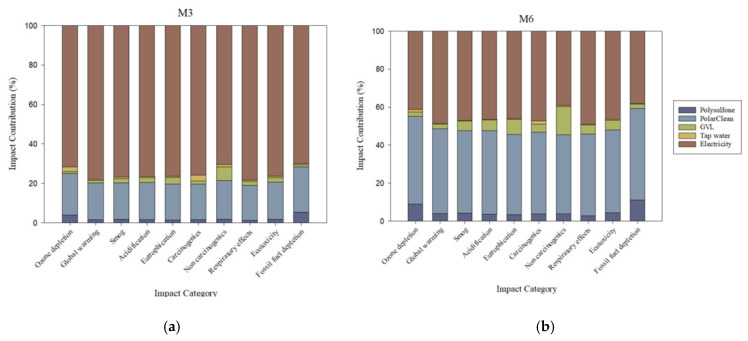
Material and energy impact contributions for the fabrication of 1 m^2^ flat sheet of (**a**) M3 and (**b**) M6, which were derived from PolarClean and GVL. Note that M3 and M6 were fabricated via DBE and SDC, respectively. Material impact contributions for membranes fabricated with DMAc and NMP are presented in Figure S2.

The relative contributions of each material to impacts from fabricating PSf-PolarClean-GVL membranes are presented in Figure 2. For comparison, relative material impact contributions from fabricating PSf-DMAc and PSf-NMP membranes are presented in Figure S2. Across all membranes, electricity followed by the solvent(s) and PSf were the main contributing parameters in all 10 categories. Among the three parameters, electricity was the most dominant in contributions, accounting for approximately 70% of impacts in M3 and at least 90% in M1 and M2 with respect to all 10 categories. Of the solvents used in DBE membranes, PolarClean exhibited the highest contributions and accounted for approximately 20% of all impacts, whereas DMAc and NMP only contributed up to 10% in most impact categories (shown in Figure S2). This disparity between the eco-friendly and conventional solvents was partially attributed to the larger quantity of PolarClean required to produce the functional unit of the membrane, though the impacts of PolarClean synthesis also played a role, as discussed later. Across M1, M2, and M3, contributions due to PSf were approximately 5% at most for each category.

When SDC was used, the additional electricity input for the equipment (0.000446 kWh) did not result in a notable increase in impacts in each category. In fact, the increased quantity of solvents caused the solvent relative contribution to rise and the electricity relative contribution to decline across all categories. Most electricity input from the fabrication process was attributed to solution mixing (28.8 kWh), indicating that the energy input was most significant for dope solution preparation and negligible for the coating process. Impacts from electricity in all categories were attributed to the global energy mix being primarily comprised of fossil fuel sources (e.g., coal, oil, and natural gas) that produce environmental emissions from extraction and combustion, as well as additional health impacts from toxic materials released during extraction [36].

### 3.3. Membrane Fabrication Impacts Comparison

**Figure 3:**
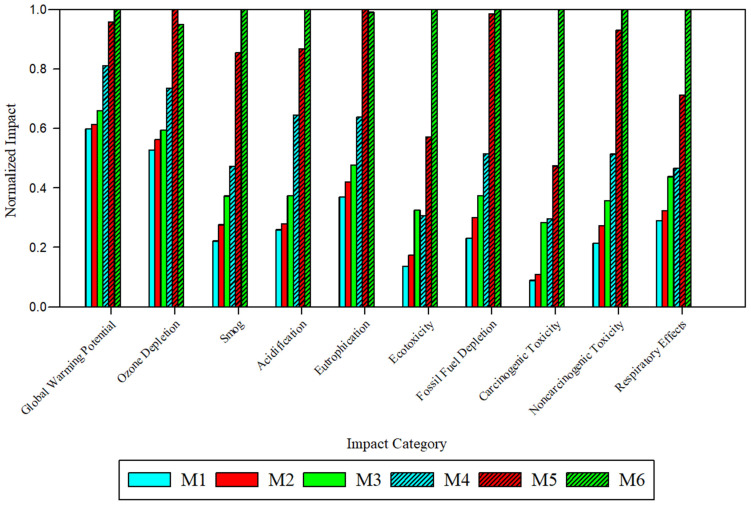
Environmental and health impacts of membrane fabrication normalized to M6 using the global average electricity process. Note that blue shades denote DMAc, red shades denote NMP, green shades denote PolarClean-GVL, and patterning denotes samples fabricated via SDC.

To compare the impacts of membrane fabrication, the global environmental and health impacts were normalized to the fabrication of 1 m^2^ of viable flat sheet membrane (Figure 3, Table S12). Across all ten impact categories, the solvent and fabrication technique type produced notable changes to the magnitude of impacts. Across all ten categories, M5 and M6 exhibited the highest impacts. M6, derived from PolarClean and GVL and fabricated via SDC, exhibited the highest impacts across 8 categories: global warming potential (2.6 × 10^−6^ kg CO_2_ eq), smog (1.7 kg O_3_ eq), acidification (0.12 kg SO_2_ eq), ecotoxicity (36.0 CTUe), fossil fuel depletion (0.13 MJ surplus), carcinogenic toxicity (56.1 CTUh), noncarcinogenic toxicity (0.04 CTUh), and respiratory effects (8.19 × 10^−6^ kg PM_2.5_ eq). M5, derived from NMP and fabricated via SDC, exhibited the highest impacts in eutrophication (1.64 × 10^−6^ kg N eq) and ozone depletion (203.1 kg CFC-_11_ eq). While M5 and M6 impacts with respect to most categories were relatively similar, M6 impacts were significantly higher than all other membranes with respect to ecotoxicity and carcinogenic toxicity.

Regarding the absolute values for impacts, the relative significance varied depending on the category and comparison with related LCA studies. Table 2 lists compiled LCA impact data between M6 and other polymeric membrane fabrication processes that were also assessed on a cradle-to-gate system. Categories such as global warming potential and respiratory effects saw relatively insignificant impact values and were several orders of magnitude lower than those reported in other studies [23,24]. Other categories, including ozone depletion and carcinogenic toxicity, exhibited relatively significant impact values and were several orders of magnitude higher than those in literature [23,24]. When analyzing the LCIA, parameters that heavily contribute to carcinogenic and ozone depletion impacts (Figure 2), including the solvent and electricity, were used in quantities ranging between approximately 0.02 and 0.81 kg and 28.8 kWh, respectively. In contrast, related studies that used the same functional unit reported electricity amounts between 117–310 kWh, thereby requiring larger amounts of extracted fossil fuels that release more carcinogenic toxins [37]. However, comparison with literature values is also not a completely straightforward measure due to the relatively small number of studies with the same functional unit and each using different membrane materials and configurations. In this case, other studies used other or additional fabrication techniques, including electrospinning and drying steps that could consume additional electricity and require longer operating times [15,24]. Other materials, including LiCl and aluminum foil (used in the electrospinning process), were included in these studies that were additional contributors to these impacts and complicate the comparison process [24].

#### 3.3.1. Impacts Comparison for Fabrication Scale-Up

Impact changes from transitioning membrane fabrication from DBE to SDC were notable in most categories. Across all membrane types, the largest increase in impacts was observed in global warming potential and carcinogenic toxicity. For PSf-DMAc and PSf-NMP membranes, carcinogenic toxicity increased by 51.2% and 85.4% when transitioning to SDC, respectively. The disparity was found to be larger for PSf-PolarClean-GVL membranes; comparing M3 and M6, impacts increased by 58.5% and 119.3% in global warming potential and carcinogenic toxicity, respectively. In other categories, the change from DBE to SDC resulted in impact increases generally between 10–30%. Together, the increases in impacts correspond to the increased material quantity, particularly increases in electricity usage from solvent production and other materials in solvent production that required fossil fuels and hazardous chemicals in upstream processes. Thus, the increased impacts for this membrane were attributed to upstream synthesis processes and not end-use properties of the membrane.

#### 3.3.2. Impacts Comparison for Solvent Type

In sharp contrast to the reported benefits of using eco-friendly solvents, the impact assessment indicated that membranes produced with PolarClean and GVL produced higher impacts across most categories (excluding eutrophication and ozone depletion) compared to those derived from DMAc and NMP. Differences in impacts were smaller between DBE-produced membranes. On average, impacts of M3 were 9.8% and 7.5% higher than those of M1 and M2, respectively. A larger disparity was found among pristine SDC membranes: impacts of M9 were 21.7% and 8.9% higher than those of M7 and M8, respectively. Thus, the higher amounts of PolarClean and GVL when using SDC enhanced the difference in impacts from conventional solvent use. In the larger context of all membrane types, however, the results pose a concern about using the PSf-PolarClean-GVL system as an alternative to conventional PSf membranes. With small to negligible GVL impact contributions, PolarClean becomes the eco-friendly solvent under scrutiny. Despite the non-hazardous properties of the final product, impacts generated by the synthesis route (PolarClean Commercial A1) produce notable relative contributions to the membrane fabrication impacts. A breakdown of material impact contributions in the PolarClean Commercial A1 synthesis route in Figure S3 shows that electricity, sulfuric acid, methanol, and acetic anhydride are major contributors. The production of these materials largely involves petrochemicals and energy-intensive operations, though the integration of cleaner energy technologies may reduce the energy footprint [40,41,42]. The conclusion of these findings is neatly summarized by Yadav et al. [14], positing that “it is important to also consider the impact of producing the green solvent; a green solvent produced via a toxic procedure cannot be expected to greatly increase the sustainability of membrane production.” Further research on the upstream solvent synthesis processes and LCA data on solvent synthesis is needed to reliably determine if eco-friendly solvents such as PolarClean and GVL can lower membrane fabrication impacts.

### 3.4. Uncertainty and Sensitivity Analyses Results

**Figure 4:**
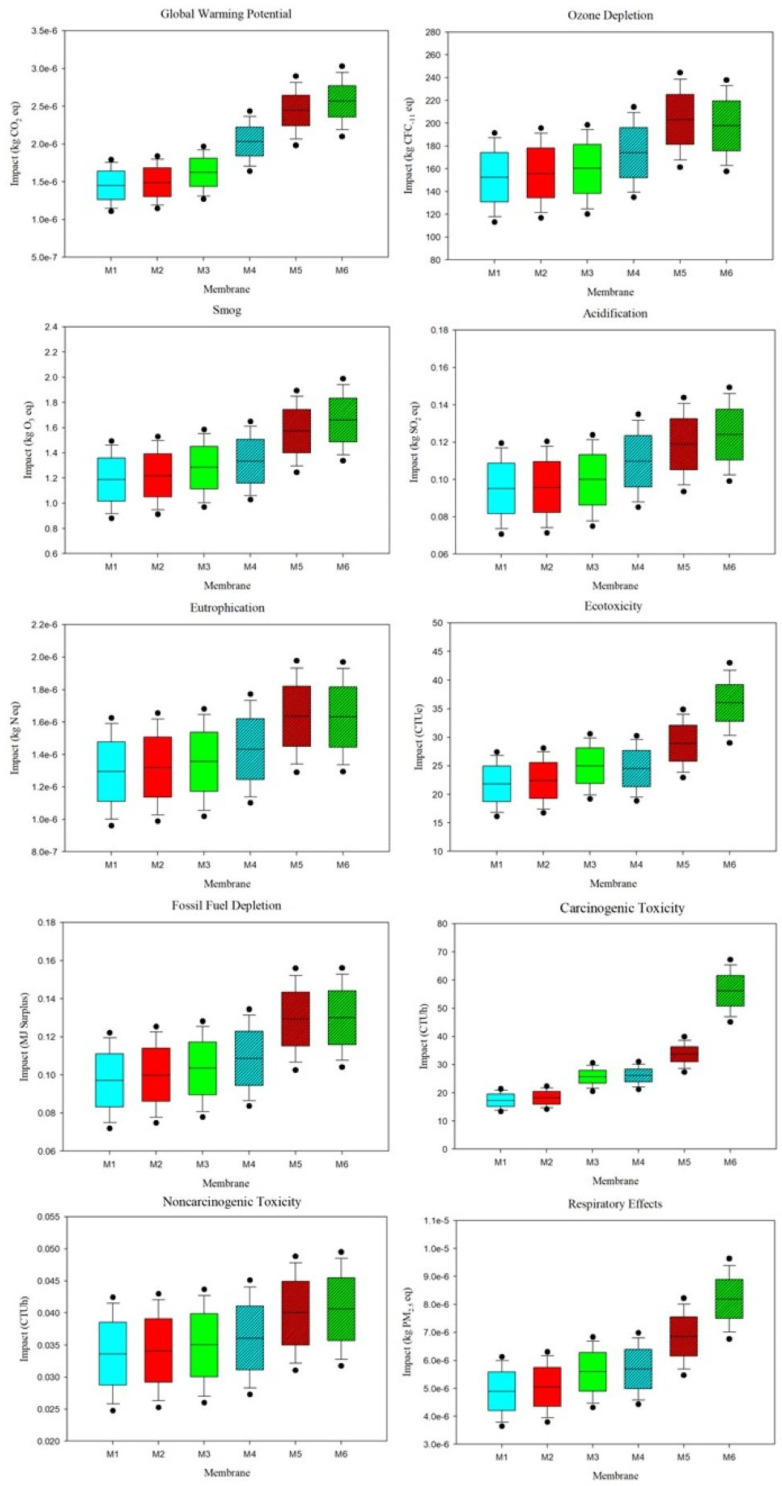
Uncertainty of impacts produced by each membrane configuration fabrication in each impact category. Dots denote the 5th and 95th percentiles. Note that blue shades denote DMAc, red shades denote NMP, green shades denote PolarClean-GVL, and patterning denotes samples fabricated via SDC.

By varying the material quantities, uncertainty in the impacts was calculated and shown in Figure 4. Uncertainty ranges in the form of minimum, median, and maximum values for each impact category are listed in Table S13. Echoing the impact assessment results, the uncertainty analysis found M5 and M6 to consistently produce significantly higher impacts than other membranes. In categories such as global warming potential, ecotoxicity, carcinogenic toxicity, and respiratory effects, the median values of M6 impacts were nearly twice as high as M1, M2, and M3. This significant disparity highlights the significant influence of PolarClean and electricity from larger parameter amounts in these categories. Across all impact categories except carcinogenic toxicity, impact variability was relatively uniform for each membrane. For carcinogenic toxicity, the interquartile range and extremes of M6 were approximately an order of magnitude larger than the ranges of the other membranes, including M4 and M5. Based on the Monte Carlo simulation approach, impact results for membranes M1-M5 with respect to carcinogenic toxicity had a high confidence interval, whereas impacts for M6 in this category and all membranes with respect to the other 9 categories were relatively lower, indicating the need for further parameter assessment to raise the confidence interval.

**Figure 5:**
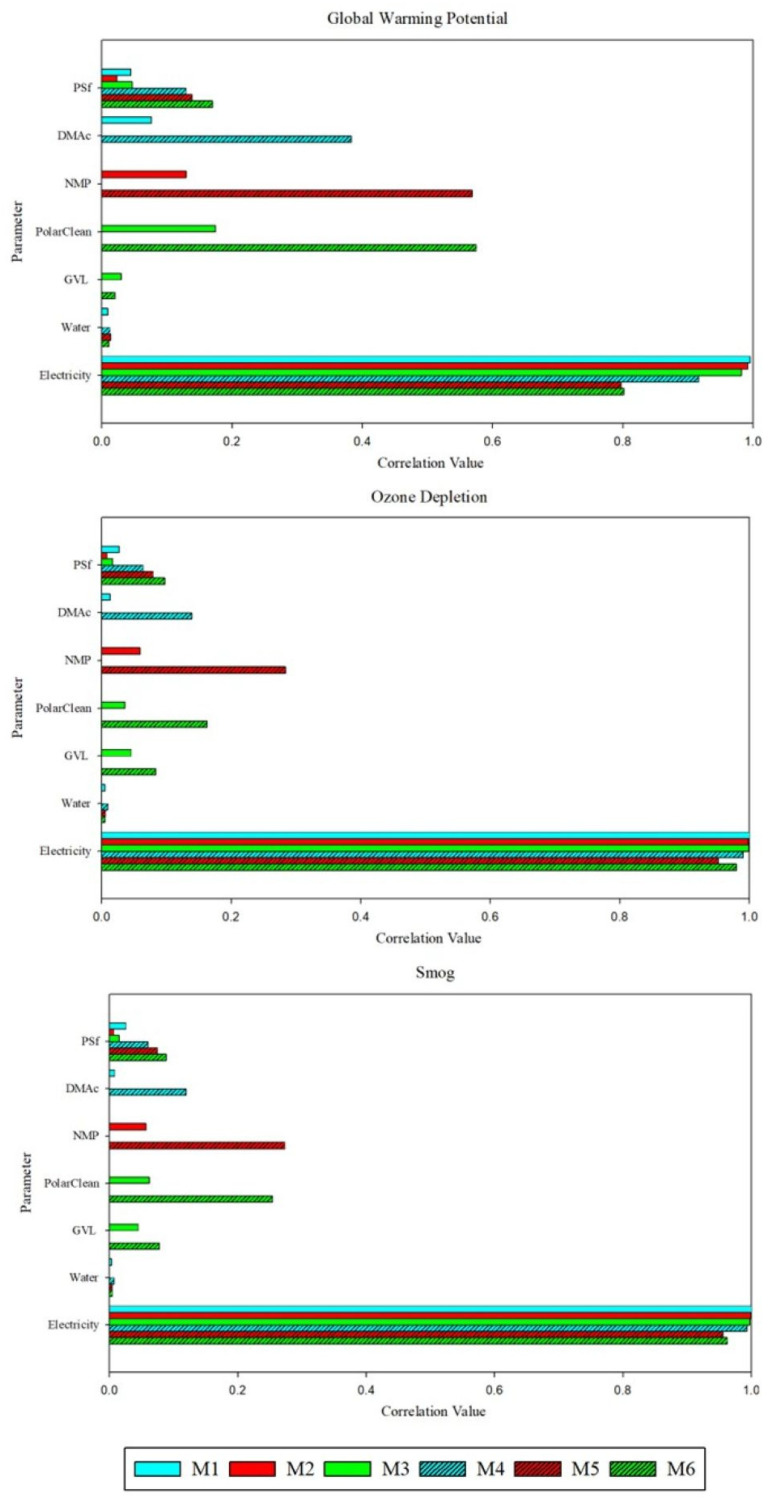
Sensitivity of impacts to membrane fabrication parameters with respect to global warming potential, ozone depletion, and smog using Spearman’s rank coefficient. Note that blue shades denote DMAc, red shades denote NMP, green shades denote PolarClean-GVL, and patterning denotes samples fabricated via SDC. Other sensitivity results are presented in Figure S4.

Sensitivity analysis of the inventory materials highlighted the role of electricity and solvent amounts in affecting the environmental and health impacts. Figure 5 shows correlation values (ρ) for the polymer, solvents, and electricity amounts calculated using Spearman’s rank coefficient for select impact categories. Membranes fabricated using both techniques were particularly sensitive to electricity. For M3, the electricity amount ρ was highest with respect to ozone depletion (0.998), acidification (0.998), eutrophication (0.998), fossil fuel depletion (0.998), noncarcinogenic toxicity (0.999), and respiratory effects (0.992); similarly, ρ for M1 and M2 was at least 0.990 in the same categories. Additionally, a distinct trend was found in impact sensitivity with respect to solvent type. For SDC membranes, the impact sensitivity of PolarClean was highest in all categories, followed by NMP, DMAc, and GVL. Among all membranes, M6 had the highest ρ, with values between 0.6 and 0.8 in each category. Thus, all impact categories were at least moderately sensitive to PolarClean for M6. Moreover, accurate assessments of PolarClean and electricity are necessary to ensure reliable LCA results. It is important to note that sensitivity analysis results are connected to the Monte Carlo uncertainty analysis. As a conservative estimate, material quantities were varied by +/−30% to account for uncertainty; however, as these technologies become more developed, more specific probability distributions would improve the uncertainty and sensitivity analyses.

### 3.5. Scenario Analysis Results

Following findings on the significant impact contribution and sensitivity of electricity, a scenario analysis was performed that substituted the regional electricity market to determine the relationship between energy grid composition and life cycle impacts. In this analysis, the global electricity market was first substituted with the US electricity market and then the Swedish (SE) electricity market. The majority of power generation in the US grid is sourced from fossil fuels (60%), while nuclear and other renewable technologies account for 19% and 21%, respectively [43]. In contrast, the Swedish grid is mainly comprised of non-fossil fuel sources, with hydro and nuclear accounting for 42% and 41% of power generation, respectively [32].

**Figure 6:**
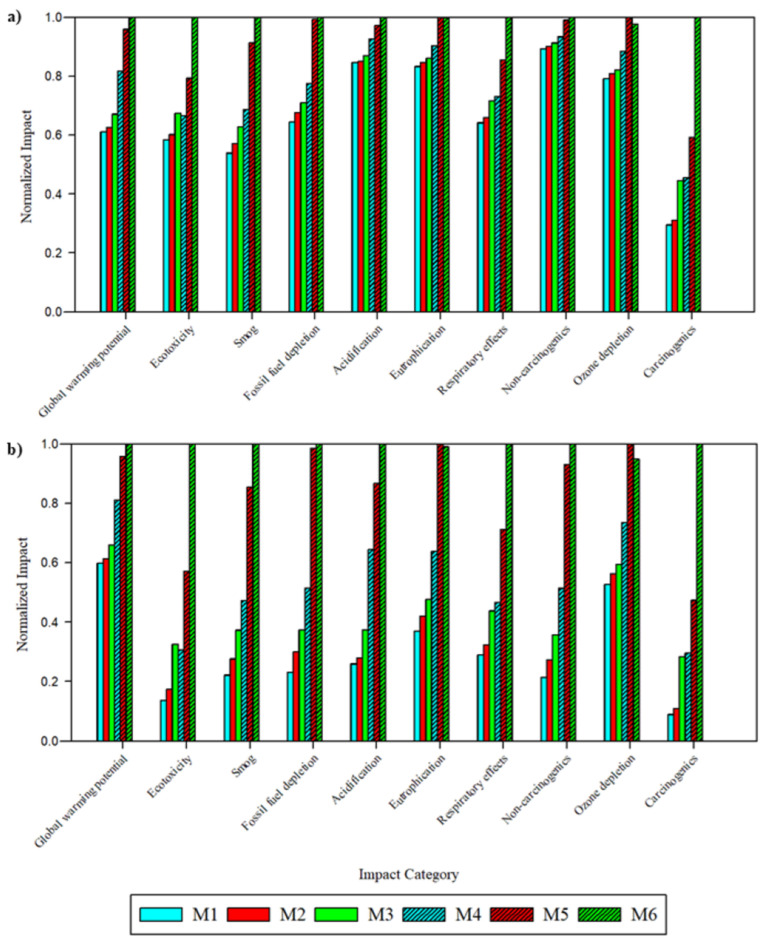
Normalized environmental and health impacts of membrane fabrication (normalized to largest impact) using (**a**) Electricity, Medium [US] and (**b**) Electricity, Medium [SE]. Note that blue shades denote DMAc, red shades denote NMP, green shades denote PolarClean-GVL, and patterning denotes incorporation of PDA-CYS-AgNP nanocomposite.

Figure 6a shows the normalized impacts of membrane fabrication using the US electricity market. Impact assessment results and percent changes relative to the global grid are presented in Table S14 and Table S15, respectively. While M6 maintained its position as the membrane type with the highest impacts in most categories, changes in impact magnitudes were observed. In comparison with global impacts, substitution with the US electricity market decreased impacts in ecotoxicity, smog, fossil fuel depletion, and carcinogenic toxicity. Pristine membranes, which had higher impact contributions from electricity, exhibited the largest declines in impacts. Smog impacts were the largest, with a −49.3% change for M3 and a −37.8% change for M6. In contrast, increased impacts were found with respect to acidification, eutrophication, and noncarcinogenic toxicity. Noncarcinogenic toxicity impact increases were the largest, ranging from 28.5% for M12 to 75.7% for M1, followed by acidification ranging between 6.4% and 68.6%. The changes in this scenario reflect differences between the fossil fuel energy mix in the US and globally. A larger reliance on natural gas than coal in the US reduces the release of heavy metals and the emission of sulfurous components that exacerbate smog formation, though higher methane emissions harm the environment in other categories [44,45].

Figure 6b shows the normalized impacts of membrane fabrication using the Swedish electricity market. Impact assessment results and percent changes relative to the global grid are presented in Table S16 and Table S17, respectively. Here, the difference in the power generation mix resulted in significant impact declines across all categories except global warming potential, which had marginal increases. For M1 and M2, impacts declined by greater than 70% in 8 categories when switching to the Swedish electricity mix. Even with a smaller impact contribution from electricity in membranes derived from PolarClean and GVL, significant impact declines were observed across most categories, including M3 and M6 decreasing in noncarcinogenic toxicity by 90.7% and 77.9%, respectively. With more electricity sourced from nuclear and hydro technologies, emissions to the environment were minimized with the extraction and combustion of fossil fuels avoided. Together with results from the US grid, the Swedish grid scenario found that the source of electricity played an important role in the impacts, and the integration of clean energy technologies can, in certain cases, significantly reduce life cycle impacts.

### 3.6. Discussion of LCA Limitations

In the review of the LCA results, it is important to discuss limitations in accuracy and potential impact reduction strategies. Of the processes identified to significantly contribute to impacts, electricity may be the easiest to modify. As shown in the scenario analysis, selecting a region with more renewable energy sources produced significant declines in most impact categories. Moreover, since all or most electricity was used for dope solution preparation, future investigations are into accelerating the mixing time to reduce electricity consumption.

As shown in the impact assessment results, the impacts of membrane fabrication with PolarClean produced were higher than those derived from DMAc and NMP. In contrast, impact assessment results from Fionah et al. [16] found differing results in which PSf membranes produced with PolarClean showed the lowest global environmental impacts in the respective comparison study. Key differences were found in the PolarClean LCI between the two studies, notably the simpler inventory in Fionah et al. [16] that consisted of isobutanol, dimethylamine, acetic anhydride, and electricity. In contrast, the inventory in this study was expanded with the inclusion of butadiene, hydrogen cyanide, sodium hydroxide, sulfuric acid, methanol, thionyl chloride, triethylamine, and toluene (Table S6), which created a disparity in impacts from producing 1 kg of PolarClean. Similar parameters between the two studies also had larger quantities in this study, including 0.174 kg of DMAc and 28.8 kWh of electricity compared to 0.0712 kg of dimethylamine and 0.1 kWh of electricity, respectively. The electricity amount of 28.8 kWh to produce 1 kg of PolarClean was an assumption in order to be uniform with the electricity amount needed to produce 1 m^2^ of PSf membrane, which was a limitation to the LCIA accuracy and highlighted the lack of available data on commercial PolarClean synthesis. As discussed in 3.3, the production of numerous hazardous, petrochemical-derived, and energy-intensive chemical feedstocks for PolarClean production raised impacts to exceed those of conventional solvents in multiple impact categories; the larger amount of electricity used to produce PolarClean in this study was another contributing factor to the disparity in results.

Together, the results of these studies highlight the importance of identifying underlying differences in LCIs and LCA methodologies when making comparisons and drawing conclusions. As different assumptions regarding synthesis routes could be used within the LCI (the synthesis route used in Fionah et al. [16] having an inventory assumed from mass balance based on Solvay’s Sustainable Portfolio Management Guide [46] and the Commercial Route A1 reported by Cseri and Szekely [26] being used for general large-scale synthesis), the studies indicate the difference in impacts is not only due to membrane fabrication scale-up but also due to nuances in solvent synthesis. As such, the results do not necessarily expand the scope of LCAs on membrane fabrication when using PolarClean as a solvent.

To leverage its eco-friendly properties while reducing impacts from its usage, alternative synthesis routes for PolarClean should be considered. Notably, Cseri and Szekely [26] developed a one-step synthesis route for PolarClean by using Michael addition reactions to build the carbon backbone and catalyzed using KO*t*Bu and phosphazene bases at 0–5 °C. This alternative route aligned with green chemistry principles with reductions of E factor and carbon intensity by 78% and 87%, respectively, compared to the patented process [26]. Moreover, an alternative route avoids the use of reagents, including methanol and DMAc, that produced notable impact contributions in this assessment.

Other limitations in the methodology were tied to the lab casting experiments for developing the material inventory and the LCA assumptions. As previously mentioned, membrane sheet thickness was not accounted for in the functional unit and the membrane samples prepared in the lab. Due to the dope solution viscosity, the thickness may have varied to an extent as each solution spread into a different surface area. As previously mentioned, the spreading of the dope solution resulted in a range of thicknesses between approximately 0.8 and 1.0 mm that influenced the LCI. In review of this trend, creating a PSf-PolarClean-GVL membrane with the same thickness as those prepared with DMAc and NMP would have lowered the inventory quantities and, subsequently, the magnitude of impacts. A sensitivity analysis of the parameter amounts due to membrane thickness could add quantified insight into the importance of controlling this dimension during membrane fabrication. Moreover, the inclusion of the ± 30% input variation in the uncertainty analysis would have also potentially covered variations in the membrane parameter inventories due to thickness differences.

Additionally, measurements of the surface defect areas on each DBE sample were done with defect areas approximated to polygonal areas. More accurate measurements of these areas would change the ratio of material lost during casting processes. The exclusion of impacts due to material transportation to the lab discounted a portion of impacts from the LCA impact assessment results. Specifying the manufacturing location of each material and calculating the distance and the transportation modes would provide further clarity on this information for impact assessment.

Furthermore, the focus of producing lab-scale membranes using SDC also created limitations on the practicality of the results when considering more realistic pilot or commercial-scale operations. While more akin to the equipment needed in commercial membrane fabrication, the SDC machinery used in this study was relatively simplistic, producing lower impacts than those expected in industry. When considering pilot or commercial scale, increased material throughput would require additional electricity for pumping solution through the slot die and additional material for tool priming, both of which would increase impacts. Additionally, the integration of a functioning R2R system to create a roll of membranes during continuous casting may require additional electricity input or potentially offer efficiency gains over DBE machinery. Nonsolvent water, which would inevitably be periodically replaced since the solvent concentration in the bath influences NIPS, would also create additional water amounts not reflected in this study [1]. When considering the materials spent as tool fluid loss during SDC, changes in tooling equipment may result in a non-linear increase in fluid tooling loss. Additionally, the ratio of material used for tooling to material that becomes a viable membrane decreases as the functional unit increases to match a more realistic commercial scale. Thus, the materials consumption and impacts during the tooling process become more negligible as the size of the membrane sheet produced increases. Further consideration is needed when translating the system design and outputs in this LCA to operations needed for more realistic fabrication scale-up. A review of scale-up factors and predefined ratios used in this study, including the dope solution-nonsolvent ratio from lab-scale experiments and scaling calculations to translate material amounts from lab-scale samples to the functional unit, would be beneficial to determine uncertainty when considering a more realistic industrial scenario.

The boundary decisions in this study also played a major role in defining impact sources and the impact types. As previously stated, a cradle-to-gate system was created for this LCA for two major reasons. First, the multitude of variables in the membrane life cycle beyond fabrication, entailing filtration application and configuration, membrane cleaning, and membrane end-of-life, would require context-specific assumptions and limit the ability to compare to existing studies [24]. This uncertainty has led LCAs on polymeric membrane fabrication to largely be bound between materials extraction and membrane production [15,23,38]. Given the common trend of a cradle-to-gate system for fabrication LCAs within the relatively sparsely reported field of polymeric membrane LCAs, following the same boundaries allowed for a fairer comparison between studies. Focusing on the impacts from the fabrication processes also fits the scope of determining strategies to modify input parameter synthesis and product fabrication to reduce impacts. Limiting the boundaries to a cradle-to-gate system created several limitations in the assessment. Without accounting for the impacts that would be produced from materials to produce a module, electricity input for pumping feed solution through the membrane, cleaning chemicals to counter fouling, and the membrane disposal process, a systematic assessment of the entire polymeric membrane lifecycle remains absent. Future studies that delve beyond the gate boundary and into the operation and end-of-life phases could identify parameters and operating steps that produce significant impacts or carry higher uncertainty that would prove useful in optimizing the rest of the life cycle.

## 4. Conclusions

From the LCA results of comparing membrane fabrication with different materials and fabrication techniques, the use of PolarClean comes under potential scrutiny as a high-impact material. Due to the high viscosity of the PSf-PolarClean-GVL dope solution, a larger material inventory to fabricate the functional unit led to higher impacts across most environmental and health categories compared to PSf membranes derived from conventional solvents. Fabricating 1 m^2^ of a PSf-PolarClean-GVL membrane using SDC required a 39.4% and 30.0% increase in dope solution volume when compared to PSf-DMAc and PSf-NMP, respectively. LCIA results found impact changes due to the solvent type and fabrication method. Using PolarClean-GVL led to increases in most impact categories: impacts of M9 were 21.7% and 8.9% higher than those of M7 and M8, respectively. Using SDC significantly increased carcinogenic toxicity impacts due to increased solvent quantity: the global warming potential and carcinogenic toxicity impacts of M6 were 58.5% and 119.3% higher compared to M3, respectively. Impacts from electricity and PolarClean due to the use of fossil fuels highlighted that impact increases were attributed to upstream parameter synthesis processes and increased impact assessment uncertainty. However, the LCA also concluded that GVL produced lower impacts compared to the other solvents. Comparison with related studies also highlighted the role of the solvent synthesis route in influencing the impact assessment and demonstrated the need for contextual discussion on the syntheses and inventories selected for developing LCAs. Several research opportunities lie ahead to determine if the impacts of fabricating PSf-PolarClean-GVL membranes can be lowered. Identifying alternative synthesis routes for PolarClean that avoid petrochemical usage and integrating renewable energy technologies in the energy grids may pave the way towards developing resilient, scalable, and more sustainable polymeric membranes for water treatment. Future LCAs that incorporate these alternative solvent synthesis routes and electricity sourced through renewable technologies may provide valuable insight into the optimized production of such membranes. Additionally, as these technologies are developed further, LCAs that explore the full life cycle of these membranes as they are applied to water treatment (e.g., using a functional unit focused on volume of water treated), including operation and end-of-life, will be beneficial.

## Figures and Tables

**Table 1:** Membrane configurations analyzed in the LCA.

Name	Solvent(s)	Fabrication Method
M1	DMAc	DBE
M2	NMP	DBE
M3	PolarClean-GVL	DBE
M4	DMAc	SDC
M5	NMP	SDC
M6	PolarClean-GVL	SDC

**Table 2:** Comparison of midpoint impacts between studies using a functional unit of producing 1 m^2^ of polymeric membrane. Note that differences in LCIA method and impact unit complicate direct impacts comparison.

Impact	Unit	M6 (This Study)	E-PDMS Membrane-Electrospraying [24]	PES Membrane [38]	PVDF-PVP-DMF MEMBRANE [39]
Global warming potential	kg CO_2_ eq	2.6 × 10^−6^	2.2 × 10^2^	N/A	7.04
Ozone Depletion	kg CFC-_11_ eq	2.0 × 10^2^	7.8 × 10^−5^	7.6 × 10^−7^	1.9 × 10^−5^
Smog	kg O_3_ eq	1.7	N/A	N/A	N/A
Acidification	kg SO_2_ eq	0.12	0.76	0.02	2.5 × 10^−2^
Eutrophication	kg N eq	1.6 × 10^−6^	6.8 × 10^−3^	N/A	1.1 × 10^−2^
Ecotoxicity	CTUe	36.0	N/A	N/A	N/A
Fossil Fuel Depletion	MJ Surplus	0.13	N/A	12.7	N/A
Carcinogenic Toxicity	CTUh	56.1	N/A	N/A	N/A
Noncarcinogenic Toxicity	CTUh	0.04	N/A	N/A	N/A
Respiratory Effects	kg PM_2.5_ eq	8.2 × 10^−6^	N/A	N/A	N/A

## Data Availability

The original contributions presented in this study are included in the article/Supplementary Material. Further inquiries can be directed to the corresponding author.

## Supplementary Materials

The following supporting information can be downloaded at: https://www.mdpi.com/article/10.3390/polym17172363/s1, Figure S1: DBE membrane samples prepared using DMAc, NMP, and PolarClean-GVL to estimate material loss due to residual dope solution left on the doctor blade and surface defects (marked on the samples); Figure S2: Material impact contributions for the fabrication of 1 m^2^ flat sheet of (a) M1, (b) M3, (c) M2, and (d) M5; Figure S3: Material impact contributions for the production of 1 kg of PolarClean via the PolarClean Commercial Route A1; Figure S4: Sensitivity of impacts to membrane fabrication parameters with respect to acidification, eutrophication, ecotoxicity, fossil fuel depletion, carcinogenic toxicity, noncarcinogenic toxicity, and respiratory effects using Spearman’s rank coefficient; Table S1: Life cycle inventory materials selected in Ecoinvent; Table S2: Life cycle inventory of PSf membranes included in the LCA; Table S3: Inventory for polysulfone (PSf); Table S4: Inventory for dimethylacetamide (DMAc); Table S5: Inventory for N-methyl-2-pyrrolidone (NMP); Table S6: Inventory for PolarClean Commercial Route A1; Table S7: Inventory for the γ-valerolactone (GVL) process; Table S8: Inventory for water; Table S9: Estimated material loss for DBE membrane configurations; Table S10: Total dope solution amounts to produce 1 m^2^ of viable flat sheet PSf membrane; Table S11: Unit impacts for producing 1 kg of each material used in PSf membrane fabrication; Table S12: Environmental and health impacts generated by membrane configurations using the global energy mix; Table S13: Environmental and health impacts generated by membrane configurations using the US energy mix, along with minimum, median, and maximum values of each impact category tabulated for the uncertainty analysis; Table S14: Environmental and health impact percent changes after substituting the global energy mix with the US energy mix; Table S15: Environmental and health impacts generated by membrane configurations using the Swedish energy mix; Table S16: Environmental and health impact percent changes after substituting the global energy mix with the Swedish energy mix.

## References

[1] Dong X., Lu D., Harris T.A.L., Escobar I.C. (2021). Polymers and Solvents Used in Membrane Fabrication: A Review Focusing on Sustainable Membrane Development. Membranes.

[2] Risk Management for Trichloroethylene (TCE). https://www.epa.gov/assessing-and-managing-chemicals-under-tsca/risk-management-trichloroethylene-tce.

[3] Dong X., Al-Jumaily A., Escobar I.C. (2018). Investigation of the Use of a Bio-Derived Solvent for Non-Solvent-Induced Phase Separation (NIPS) Fabrication of Polysulfone Membranes. Membranes.

[4] Cardoso A.P., Giacobbo A., Bernardes A.M., Ferreira C.A. (2024). Green solvent γ-Valerolactone as a sustainable alternative for the production of polymeric membranes for pharmaceutically active compounds (PhACs) removal from water. J. Environ. Chem. Eng..

[5] Dong X., Shannon H.D., Parker C., De Jesus S., Escobar I.C. (2019). Comparison of two low-hazard organic solvents as individual and cosolvents for the fabrication of polysulfone membranes. AIChE J..

[6] Lu D., Babaniamansour P., Williams A., Opfar K., Nurick P., Escobar I.C. (2022). Fabrication and evaporation time investigation of water treatment membranes using green solvents and recycled polyethylene terephthalate. J. Appl. Polym. Sci..

[7] Dong X., Jeong T.J., Kline E., Banks L., Grulke E., Harris T., Escobar I.C. (2020). Eco-friendly solvents and their mixture for the fabrication of polysulfone ultrafiltration membranes: An investigation of doctor blade and slot die casting methods. J. Membr. Sci..

[8] Lu D., Jung K., Shim J.Y., Harris T.A.L., Escobar I.C. (2024). Manufacturing supported loose-nanofiltration polymeric membranes with eco-friendly solvents on an R2R System. npj Clean Water.

[9] Chede S., Griffiths P., Escobar I.C., Harris T.A.L. (2017). Does casting method matter in filtration membranes? A comparison in performance between doctor blade and slot-die extruded polymeric membranes. J. Appl. Polym. Sci..

[10] Azapagic A. (1999). Life cycle assessment and its application to process selection, design and optimisation. Chem. Eng. J..

[11] (2006). Environmental management—Life cycle assessment—Principles and framework.

[12] (2006). Environmental management—Life cycle assessment—Requirements and guidelines.

[13] Guinée J.B., Heijungs R., Huppes G., Zamagni A., Masoni P., Buonamici R., Ekvall T., Rydberg T. (2010). Life Cycle Assessment: Past, Present, and Future. Environ. Sci. Technol..

[14] Yadav P., Ismail N., Essalhi M., Tysklind M., Athanassiadis D., Tavajohi N. (2021). Assessment of the environmental impact of polymeric membrane production. J. Membr. Sci..

[15] Hong S.U., Wang Y., Soh L.S., Yong W.F. (2023). Are green solvents truly green? Integrating life cycle assessment and techno-economic analysis for sustainable membrane fabrication. Green Chem..

[16] Fionah A., Oluk I., Brady L., Byrne D.M., Escobar I.C. (2024). Performance and Environmental Assessment of Biochar-Based Membranes Synthesized from Traditional and Eco-Friendly Solvents. Membranes.

[17] Moradi K., Firouzjaei M.D., Elliott M., Sadrzadeh M. (2024). Lifecycle assessment of membrane synthesis for the application of thermo-osmotic energy conversion process. Case Stud. Chem. Environ. Eng..

[18] Thi H.Y.N., Kim S., Nguyen B.T.D., Lim D., Kumar S., Lee H., Szekely G., Kim J.F. (2022). Closing the Sustainable Life Cycle Loop of Membrane Technology via a Cellulose Biomass Platform. ACS Sustain. Chem. Eng..

[19] Zhao G., Hughes D., Beynon D., Wei Z., Watson T., Tsoi W.C., Baker J. (2024). Perovskite photovoltaics for aerospace applications–life cycle assessment and cost analysis. Sol. Energy.

[20] Espinosa N., García-Valverde R., Urbina A., Krebs F.C. (2011). A life cycle analysis of polymer solar cell modules prepared using roll-to-roll methods under ambient conditions. Sol. Energy Mater. Sol. Cells.

[21] Espinosa N., García-Valverde R., Urbina A., Lenzmann F., Manceau M., Angmo D., Krebs F.C. (2012). Life cycle assessment of ITO-free flexible polymer solar cells prepared by roll-to-roll coating and printing. Sol. Energy Mater. Sol. Cells.

[22] Vesce L., Stefanelli M., Rossi F., Castriotta L.A., Basosi R., Parisi M.L., Sinicropi A., Carlo A.D. (2024). Perovskite solar cell technology scaling-up: Eco-efficient and industrially compatible sub-module manufacturing by fully ambient air slot-die/blade meniscus coating. Prog. Photovolt. Res. Appl..

[23] Prézélus F., Tiruta-Barna L., Guigui C., Remigy J.-C. (2021). A generic process modelling—LCA approach for UF membrane fabrication: Application to cellulose acetate membranes. J. Membr. Sci..

[24] Hu X., Guo J., An A.K., Chopra S.S. (2023). Electrospun nanofibrous membranes for membrane distillation application—A dynamic life cycle assessment (dLCA) approach. Water Res..

[25] Frischknecht R., Jungbluth N., Althaus H.-J., Doka G., Dones R., Heck T., Hellweg S., Hischier R., Nemecek T., Rebitzer G. (2005). The ecoinvent Database: Overview and Methodological Framework (7 pp). Int. J. Life Cycle Assess..

[26] Cseri L., Szekely G. (2019). Towards cleaner PolarClean: Efficient synthesis and extended applications of the polar aprotic solvent methyl 5-(dimethylamino)-2-methyl-5-oxopentanoate. Green Chem..

[27] Han J., Son M., Kang D. (2022). Process design and environmental analysis for catalytic production of gamma-valerolactone from Kenaf. J. Ind. Eng. Chem..

[28] Bare J. (2011). TRACI 2.0: The tool for the reduction and assessment of chemical and other environmental impacts 2.0. Clean Technol. Environ. Policy.

[29] Arvidsson R., Svanström M., Harvey S., Sandén B.A. (2021). Life-cycle impact assessment methods for physical energy scarcity: Considerations and suggestions. Int. J. Life Cycle Assess..

[30] Erhart S., Erhart K. (2023). Environmental ranking of European industrial facilities by toxicity and global warming potentials. Sci. Rep..

[31] Gronlund C.J., Humbert S., Shaked S., O’nEill M.S., Jolliet O. (2014). Characterizing the burden of disease of particulate matter for life cycle impact assessment. Air Qual. Atmos. Heal..

[32] Shanmugam K., Gadhamshetty V., Yadav P., Athanassiadis D., Tysklind M., Upadhyayula V.K.K. (2019). Advanced High-Strength Steel and Carbon Fiber Reinforced Polymer Composite Body in White for Passenger Cars: Environmental Performance and Sustainable Return on Investment under Different Propulsion Modes. ACS Sustain. Chem. Eng..

[33] Liu W., Canfield N. (2012). Development of thin porous metal sheet as micro-filtration membrane and inorganic membrane support. J. Membr. Sci..

[34] Ismail A., Lai P. (2003). Effects of phase inversion and rheological factors on formation of defect-free and ultrathin-skinned asymmetric polysulfone membranes for gas separation. Sep. Purif. Technol..

[35] Chung T.-S., Kafchinski E.R. (1997). The effects of spinning conditions on asymmetric 6FDA/6FDAM polyimide hollow fibers for air separation. J. Appl. Polym. Sci..

[36] Karstensen J., Peters G. (2017). Distributions of carbon pricing on extraction, combustion and consumption of fossil fuels in the global supply-chain. Environ. Res. Lett..

[37] Hilakivi-Clarke L., Jolejole T.K., da Silva J.L., Andrade F.d.O., Dennison G., Mueller S. (2025). Aromatics from fossil fuels and breast cancer. iScience.

[38] Zuin S., Scanferla P., Brunelli A., Marcomini A., Wong J.E., Wennekes W., Genné I. (2013). Layer-by-Layer Deposition of Titanium Dioxide Nanoparticles on Polymeric Membranes: A Life Cycle Assessment Study. Ind. Eng. Chem. Res..

[39] Bhalani D.V., Zhang Q., Yang Y., Jewrajka S.K., Shen J. (2024). Fabrication of superhydrophilic membranes for oil-water separation: A life cycle assessment study. ChemRxiv..

[40] Ma L., Li J., Zhanfg H., Xu L. (2025). Life Cycle Assessment and Economic Analysis of Aspirin Production. Sustain. Chem. Pharm..

[41] Capello C., Wernet G., Sutter J., Hellweg S., Hungerbühler K. (2009). A comprehensive environmental assessment of petrochemical solvent production. Int. J. Life Cycle Assess..

[42] Kiss A.A., Bildea C.S., Verheijen P.J.T. (2006). Optimization studies in sulfuric acid production. Comput. Aided Chem. Eng..

[43] What Is U.S. Electricity Generation by Energy Source?. https://www.eia.gov/tools/faqs/faq.php?id=427&t=3.

[44] Mira-Salama D., Grüning C., Jensen N., Cavalli P., Putaud J.-P., Larsen B., Raes F., Coe H. (2008). Source attribution of urban smog episodes caused by coal combustion. Atmos. Res..

[45] Howarth R.W. (2014). A bridge to nowhere: Methane emissions and the greenhouse gas footprint of natural gas. Energy Sci. Eng..

[46] Sustainable Portfolio Management Guide: Driving Long-Term Sustainable Growth. https://www.solvay.com/sites/g/files/srpend221/files/2022-07/Sustainable%20Portfolio%20Management%20%28SPM%29%20Guide.pdf.

