# Supplementary material for: Comparative Life Cycle Assessment for the Fabrication of Polysulfone Membranes Using Slot Die Coating as a Scalable Fabrication Technique
Source: Polymers (Basel). 2025 Aug 30;17(17):2363. doi: 10.3390/polym17172363 (PMC12431056; doi:10.3390/polym17172363)
Supplement: Supplementary file 1 [file polymers-17-02363-s001.zip › polymers-3822882-supplementary.pdf]

Comparative Life Cycle Assessment for the Fabrication of Polysulfone Membranes Using Slot Die Coating as a Scalable Fabrication Technique

David Lu [1], Isaac Oluk [2], Minwoo Jung [3], Sophia Tseng [1], Diana M. Byrne [2], Tequila A. L. Harris [3] and Isabel C. Escobar [1,*]

[1] Department of Chemical and Materials Engineering, University of Kentucky, Lexington, KY 40506, USA

[2] Department of Civil Engineering, University of Kentucky, Lexington, KY 40506, USA

[3] George W. Woodruff School of Mechanical Engineering, Georgia Institute of Technology, Atlanta, GA 30332, USA

Supporting Information

Table S1. Life cycle inventory materials selected in Ecoinvent. *denotes custom-built processes based on reported literature. Processes for PolarClean and GVL synthesis are described in Table S6 and S7, respectively.

Material	Component
Polysulfone (GLO)| market for | APOS, U	Polymer
Dimethylacetamide (GLO)| market for | APOS, U	Solvent
N-methyl-2-pyrrolidone (RoW)| market for| APOS, U	Solvent
PolarClean Commercial A1*	Solvent
GVL Unit Process*	Solvent
Tap water (GLO)| market group for | APOS, U	Nonsolvent

Table S2. Life Cycle Inventory of PSf Membranes included in the LCA.

Material	Units	M1	M2	M3	M4	M5	M6
Polysulfone	kg/m^2	0.034	0.029	0.056	0.155	0.166	0.216
DMAc	kg/m^2	0.165	0	0	0.758	0	0
NMP	kg/m^2	0	0.143	0	0	0.812	0
PolarClean	kg/m^2	0	0	0.206	0	0	0.792
GVL	kg/m^2	0	0	0.069	0	0	0.264

Water	kg/m^2	104.544	104.544	104.544	104.545	104.545	104.545
Electricity	kWh	28.8	28.8	28.8	28.801	28.801	28.801

Table S3. Inventory for polysulfone (PSf). Applicable Ecoinvent process: polysulfone (GLO)| polysulfone production for membrane filtration production | APOS, U.

**Inputs**			**Outputs**		
Nature	*Amount*	*Unit*	*Product*	*Amount*	*Unit*
Oxygen	0.404	kg	Polysulfone	1	kg
Water, cooling	0.024	m^3			
Water	0.012	m^3	*Emissions to Air*	*Amount*	*Unit*
			Phenol	0.001	kg
Technosphere	*Amount*	*Unit*	Phenol, 2,4-dichloro-	0.002	kg
2,4-dichlorophenol	0.932	kg	Water/m^3	0.012	m^3
Benzene	0.999	kg			
Bisphenol A, powder	0.360	kg			
Chemical factory, organics	3.999E-10	p			
Electricity, medium voltage	0.485	kWh			
Heat, from steam, in chemical industry (RER)	0.330	MJ			

Heat, from steam, in chemical industry (RoW)	1.669	MJ	

Table S4. Inventory for dimethylacetamide (DMAc). Applicable Ecoinvent process: dimethylacetamide (GLO)| production | APOS, U.

**Inputs**			**Outputs**		
Nature	*Amount*	*Unit*	*Product*	*Amount*	*Unit*
Water, cooling, unspecified natural origin	0.016	m^3	Dimethylacetamide	1	kg
Water, river	0.001	m^3			
Water, well	0.001	m^3	*Emissions to Air*	*Amount*	*Unit*
			Acetic acid	0.001	kg
Technosphere	*Amount*	*Unit*	Carbon dioxide, fossil	0.091	kg
Acetic acid	0.726	kg	Dimethylamine	0.001	kg
Chemical factory, organics	3.999	p	Nitrogen, atmospheric	0.019	kg
Dimethylamine (RER)	0.095	kg	Water/m^3	0.001	m^3
Dimethylamine (RoW)	0.095	kg			

Electricity, medium voltage	0.416	kWh	*Emissions to Water*	*Amount*	*Unit*
Heat, district or industrial, natural gas	2.150	MJ	Acetic acid	0.003	kg
Heat, from steam, in chemical industry (RER)	0.033	MJ	BOD5, biological oxygen demand	0.01	kg
Heat, from steam, in chemical industry (RoW)	0.167	MJ	COD, chemical oxygen demand	0.002	kg
Nitrogen, liquid (RER)	0.004	kg	Dimethylamine	0.003	kg
Nitrogen, liquid (RoW)	0.014	kg	DOC, dissolved organic carbon water	0.001 0.017	kg
Tap water	0.026	kg			
			TOC, total organic carbon	0.003	kg
			Water	0.017	m^3
			Waste and Emissions to Treatment	*Amount*	*Unit*

	Wastewater, average (CH)	9.127	m^3
	Wastewater, average (Europe without Switzerland)	8.437	m^3
	Wastewater, average (RoW)	1.765	m^3

Table S5. Inventory for N-methyl-2-pyrrolidone (NMP). Applicable Ecoinvent process: N-methyl-2-pyrrolidone (RoW)| production | APOS, U.

**Inputs**			**Outputs**		
Nature	*Amount*	*Unit*	*Product*	*Amount*	*Unit*
Water, cooling, unspecified natural origin	0.016	m^3	N-methyl-2-pyrrolidone	1	kg
Water, river	0.001	m^3			
Water, well	0.001	m^3	*Emissions to Air*	*Amount*	*Unit*
			Carbon dioxide, fossil	0.052	kg
Technosphere	*Amount*	*Unit*	Methylamine	0.003	kg
Butyrolactone	0.895	kg	Nitrogen, atmospheric	0.019	kg

Chemical factory, organics	3.999	p	Water/m^3	0.001	m^3
Electricity, medium voltage (AU)	0.005	kWh			
Electricity, medium voltage (NZ)	0.001	kWh	*Emissions to Water*	*Amount*	*Unit*
Electricity, medium voltage (RAF)	0.016	kWh	BOD5, biological oxygen demand	0.002	kg
Electricity, medium voltage (RAS)	0.232	kWh	Butyrolactone	0.001	kg
Electricity, medium voltage (RLA)	0.031	kWh	COD, chemical oxygen demand	0.002	kg
Electricity, medium voltage (RNA)	0.109	kWh	DOC, dissolved organic carbon	0.001	kg
Electricity, medium voltage (RoW)	4.137	kWh	Water	0.017	m^3
Electricity, medium voltage (RU)	0.022	kWh			
Heat, district or industrial, natural gas (CA-QC)	0.037	MJ			

Heat, district or industrial, natural gas (RoW)	2.113	MJ	*Waste and Emissions to Treatment*	*Amount*	*Unit*
Heat, from steam, in chemical industry	0.199	MJ	Wastewater, average	2.699	m^3
Methylamine	0.969	kg			
Nitrogen, liquid	0.019	kg			
Tap water (CA-QC)	4.872	kg			
Tap water (RoW)	0.026	kg			

Table S6. Inventory for PolarClean Commercial Route A1.

**Inputs**			**Outputs**		
Nature	*Amount*	*Unit*	*Product*	*Amount*	*Unit*
Butadiene	0.054	kg	PolarClean	0.983	kg
Hydrogen cyanide	0.054	kg			
Sodium hydroxide, without water, in 50% solution state	0.039	kg			
Sulfuric acid	0.098	kg			
Water, deionized, from tap water, at user	0.036	kg			

Acetic anhydride	0.225	kg	
Methanol, from biomass	0.641	kg	
Thionyl chloride	0.238	kg	
Dimethylacetamide	0.174	kg	
Triethyl amine	0.126	kg	
Toluene, liquid	0.092	kg	
Electricity, high voltage	28.8	kWh	

Table S7. Inventory for γ-valerolactone (GVL) process.

**Inputs**			**Outputs**		
Nature	*Amount*	*Unit*	*Product*	*Amount*	*Unit*
Kenaf fiber	4.601	kg	GVL	1	kg
Sulfuric acid	0.046	kg			
Packing, lime product	0.035	kg	*Waste and Emissions to Treatment*	*Amount*	*Unit*
Water, deionized, from tap water, at user	1.872	kg	Waste gypsum	0.081	kg
Hydrogen, liquid	0.016	kg	Wood ash mixture	0.286	kg

Table S8. Inventory for water. Applicable Ecoinvent process: tap water (RoW) | tap water production, direct filtration treatment | APOS, U.

Inputs			Outputs		
Nature	*Amount*	*Unit*	*Product*	*Amount*	*Unit*
Water, river	0.001	m^3	Tap water	1	kg
Technosphere	*Amount*	*Unit*	*Waste and Emissions to Treatment*	*Amount*	*Unit*
Chlorine, liquid	3.209	kg	Wastewater, unpolluted	1.285	m^3
Diesel, burned in building machine	6.640	MJ			
Electricity, medium voltage (NZ)	3.517	kWh			
Electricity, medium voltage (Canada without Quebec)	6.767	kWh			
Electricity, medium voltage (RAF)	6.810	kWh			
Electricity, medium voltage (RAS)	1.125	kWh			
Electricity, medium voltage (RLA)	1.615E-4	kWh			

Electricity, medium voltage (RoW)	2.166	kWh	
Electricity, medium voltage (RU)	2.881	kWh	
Electricity, medium voltage (US)	6.530	kWh	
Heat, district or industrial, natural gas	1.160	MJ	
Water works, capacity 1.1E10L/year	1.521	p	

Table S9. Estimated material loss for DBE membrane configurations.

	**PSf-DMAc (M1, M4)**	**PSf-NMP (M2, M5)**	**PSf-PolarClean-GVL (M3, M6)**
Total defective area (cm^2)	0.013	0.001	0
Total defect dope solution volume (mL)	2.098	0.257	0
Residual dope solution volume (mL)	0.489	0.407	0.396
Total dope solution volume (mL)	2.587	0.666	0.396
Material loss factor	1.517	1.133	1.079

Table S10. Total dope solution amounts to produce 1 m^2 of viable flat sheet PSf membrane.

**Fabrication Technique**	**PSf-DMAc (kg)**	**PSf-NMP (kg)**	**PSf-PolarClean-GVL (kg)**
DBE (M1-M3)	0.131	0.152	0.307
SDC (M4-M6)	0.913	0.979	1.273

Table S11. Unit impacts for producing 1 kg of each material used in PSf membrane fabrication.

Impact category	Unit	Polysulfone	DMAc	NMP	Polarclean	GVL	Tap Water	Electricity
Global warming	kg CO2 eq	7.912	3.123	8.184	15.67	3.671	6.904×10^{-4}	0.596
Ozone depletion	kg CFC-11 eq	1.267×10^{-6}	7.312×10^{-7}	1.145×10^{-6}	1.195×10^{-6}	2.388×10^{-7}	1.743×10^{-10}	3.707×10^{-8}
Smog	kg O3 eq	0.452	0.164	0.431	0.374	0.403	4.166×10^{-5}	1.255×10^{-2}
Acidification	kg SO2 eq	3.283×10^{-2}	1.372×10^{-2}	3.729×10^{-2}	2.349×10^{-2}	3.476×10^{-2}	3.195×10^{-6}	4.211×10^{-4}
Eutrophication	kg N eq	2.985×10^{-2}	1.931×10^{-2}	2.827×10^{-2}	1.410×10^{-2}	5.154×10^{-2}	2.663×10^{-6}	9.248×10^{-5}
Ecotoxicity	CTUe	63.18	24.97	57.38	27.46	49.01	7.415×10^{-3}	0.269
Fossil fuel depletion	MJ surplus	19.90	10.81	18.99	45.73	5.814	5.448×10^{-4}	1.749
Carcinogenics	CTUh	4.394×10^{-7}	1.555×10^{-7}	3.814×10^{-7}	2.243×10^{-7}	3.219×10^{-7}	1.891×10^{-10}	2.929×10^{-9}
Noncarcinogenics	CTUh	2.099×10^{-6}	9.576×10^{-7}	2.265×10^{-6}	1.602×10^{-6}	6.380×10^{-6}	2.939×10^{-10}	1.217×10^{-8}
Respiratory effects	kg PM2.5 eq	8.683×10^{-3}	2.717×10^{-3}	7.189×10^{-3}	3.551×10^{-3}	9.436×10^{-3}	1.085×10^{-6}	3.124×10^{-5}

Table S12. Environmental and health impacts generated by membrane configurations using the global energy mix.

Impact Category	Unit	M1	M2	M3	M4	M5	M6
Global warming	kg CO2 eq	1.447×10^{-6}	1.489×10^{-6}	1.617×10^{-6}	2.037×10^{-6}	2.444×10^{-6}	2.562×10^{-6}
Ozone depletion	kg CFC-11 eq	1.52×10^{2}	1.559×10^{2}	1.593×10^{2}	1.746×10^{2}	2.031×10^{2}	1.976×10^{2}
Smog	kg O3 eq	1.185	1.218	1.277	1.338	1.571	1.660
Acidification	kg SO2 eq	9.495×10^{-2}	9.571×10^{-2}	9.942×10^{-2}	1.101×10^{-1}	1.188×10^{-1}	1.24×10^{-1}
Eutrophication	kg N eq	1.291×10^{-6}	1.319×10^{-6}	1.350×10^{-6}	1.437×10^{-6}	1.635×10^{-6}	1.630×10^{-6}
Ecotoxicity	CTUe	21.74	22.36	24.87	24.56	28.91	35.99
Fossil fuel depletion	MJ surplus	9.685×10^{-2}	9.979×10^{-2}	1.030×10^{-1}	1.090×10^{-1}	1.293×10^{-1}	1.299×10^{-1}
Carcinogenic toxicity	CTUh	17.29	18.16	25.57	26.13	33.67	56.08
Noncarcinogenic toxicity	CTUh	3.354×10^{-2}	3.406×10^{-2}	3.482×10^{-2}	3.621×10^{-2}	3.994×10^{-2}	4.057×10^{-2}
Respiratory effects	Kg $PM_{2.5}$ eq	4.881×10^{-6}	5.039×10^{-6}	5.572×10^{-6}	5.706×10^{-6}	6.847×10^{-6}	8.187×10^{-6}

Table S13. Environmental and health impacts generated by membrane configurations using the US energy mix, along with minimum, median, and maximum values of each impact category tabulated for the uncertainty analysis.

	**M1**	**M2**	**M3**	**M4**	**M5**	**M6**
	Global Warming Potential (kg CO_2 eq)					
Min	1.028×10^{-6}	1.054×10^{-6}	1.156×10^{-6}	1.450×10^{-6}	1.754×10^{-6}	1.833×10^{-6}
Median	1.451×10^{-6}	1.489×10^{-6}	1.624×10^{-6}	2.034×10^{-6}	2.445×10^{-6}	2.567×10^{-6}
Max	1.870×10^{-6}	1.922×10^{-6}	2.078×10^{-6}	2.622×10^{-6}	3.149×10^{-6}	3.294×10^{-6}
	Ozone Depletion (kg CFC_{-11} eq)					
Min	107.03	110.01	113.07	123.94	144.99	141.27
Median	152.37	155.84	160.21	173.89	203.24	197.68
Max	197.13	201.71	205.67	225.44	262.53	254.88
	Smog (kg O_3 eq)					
Min	0.83	0.86	0.91	0.95	1.12	1.19
Median	1.19	1.22	1.28	1.33	1.57	1.66
Max	1.54	1.58	1.65	1.73	2.03	2.14
	Acidification (kg SO_2 eq)					
Min	0.07	0.07	0.07	0.08	0.08	0.09
Median	0.10	0.10	0.10	0.11	0.12	0.12
Max	0.12	0.12	0.13	0.14	0.15	0.16
	Eutrophication (kg N eq)					
Min	9.122×10^{-7}	9.328×10^{-7}	9.603×10^{-7}	1.022×10^{-6}	1.165×10^{-6}	1.167×10^{-6}
Median	1.294×10^{-6}	1.318×10^{-6}	1.357×10^{-6}	1.431×10^{-6}	1.637×10^{-6}	1.632×10^{-6}
Max	1.675×10^{-6}	1.705×10^{-6}	1.742×10^{-6}	1.856×10^{-6}	2.111×10^{-6}	2.099×10^{-6}

	Fossil Fuel Depletion (MJ surplus)					
Min	6.810×10^{-2}	7.036×10^{-2}	7.311×10^{-2}	7.728×10^{-2}	9.220×10^{-2}	9.275×10^{-2}
Median	9.708×10^{-2}	9.976×10^{-2}	1.035×10^{-1}	1.086×10^{-1}	1.294×10^{-1}	1.300×10^{-1}
Max	1.256×10^{-1}	1.292×10^{-1}	1.329×10^{-1}	1.410×10^{-1}	1.673×10^{-1}	1.676×10^{-1}
	Ecotoxicity (CTUe)					
Min	15.3	15.8	17.7	17.4	20.6	25.7
Median	21.8	22.4	25.0	24.5	28.9	36.0
Max	28.2	29.0	32.1	31.7	37.4	46.3
	Carcinogenic Toxicity (CTUh)					
Min	12.3	12.8	18.3	18.6	24.2	39.9
Median	17.3	18.2	25.6	26.1	33.7	56.2
Max	22.3	23.4	32.9	33.6	43.4	71.9
	Noncarcinogenic Toxicity (CTUh)					
Min	2.355×10^{-2}	2.401×10^{-2}	2.465×10^{-2}	2.561×10^{-2}	2.832×10^{-2}	2.882×10^{-2}
Median	3.362×10^{-2}	3.404×10^{-2}	3.505×10^{-2}	3.605×10^{-2}	4.002×10^{-2}	4.060×10^{-2}
Max	4.354×10^{-2}	4.413×10^{-2}	4.505×10^{-2}	4.685×10^{-2}	5.174×10^{-2}	5.240×10^{-2}
	Respiratory Effects (Kg $PM_{2.5}$ eq)					
Min	3.440×10^{-6}	3.558×10^{-6}	3.974×10^{-6}	4.052×10^{-6}	4.900×10^{-6}	5.879×10^{-6}
Median	4.890×10^{-6}	5.039×10^{-6}	5.604×10^{-6}	5.689×10^{-6}	6.852×10^{-6}	8.193×10^{-6}
Max	6.327×10^{-6}	6.518×10^{-6}	7.172×10^{-6}	7.366×10^{-6}	8.848×10^{-6}	1.051×10^{-5}

Table S14. Environmental and health impact percent changes after substituting the global energy mix with the US energy mix.

**Impact Category**	**Unit**	**M1**	**M2**	**M3**	**M4**	**M5**	**M6**
Global warming	kg CO2 eq	21.588	20.988	19.327	15.341	12.784	12.195
Ozone depletion	kg CFC-11 eq	28.191	27.493	26.910	24.551	21.107	21.690
Smog	kg O3 eq	-53.145	-51.695	-49.308	-47.062	-40.077	-37.941
Acidification	kg SO2 eq	68.564	68.016	65.480	59.154	54.791	52.489
Eutrophication	kg N eq	33.111	32.425	31.684	29.746	26.152	26.235
Fossil fuel depletion	MJ surplus	-38.166	-37.042	-35.902	-33.899	-28.595	-28.453
Ecotoxicity	CTUe	-8.142	-7.915	-7.117	-7.206	-6.122	-4.919
Carcinogenic toxicity	CTUh	-5.979	-5.691	-4.042	-3.955	-3.070	-1.843
Noncarcinogenic toxicity	CTUh	75.654	74.500	72.870	70.067	63.529	62.546
Respiratory effects	Kg $PM_{2.5}$ eq	21.349	20.676	18.700	18.262	15.218	12.728

Table S15. Environmental and health impacts generated by membrane configurations using the Swedish energy mix.

**Impact Category**	**Unit**	**M1**	**M2**	**M3**	**M4**	**M5**	**M6**
Global warming	kg CO2 eq	1.660×10^{-6}	1.701×10^{-6}	1.829×10^{-6}	2.249×10^{-6}	2.656×10^{-6}	2.774×10^{-6}

Ozone depletion	kg CFC-11 eq	56.83	60.69	64.06	79.37	1.079×10^{2}	1.024×10^{2}
Smog	kg O3 eq	1.354×10^{-1}	1.687×10^{-1}	2.276×10^{-1}	2.886×10^{-1}	5.218×10^{-1}	6.103×10^{-1}
Acidification	kg SO2 eq	1.021×10^{-2}	1.098×10^{-2}	1.468×10^{-2}	2.532×10^{-2}	3.408×10^{-2}	3.929×10^{-2}
Eutrophication	kg N eq	2.020×10^{-7}	2.293×10^{-7}	2.602×10^{-7}	3.481×10^{-7}	5.457×10^{-7}	5.405×10^{-7}
Ecotoxicity	CTUe	22.49	28.71	53.78	50.71	94.21	16.49
Fossil fuel depletion	MJ surplus	9.961×10^{-3}	1.290×10^{-2}	1.607×10^{-2}	2.215×10^{-2}	4.238×10^{-2}	4.302×10^{-2}
Carcinogenic toxicity	CTUh	38.21	46.94	121.1	126.7	202.1	426.2
Noncarcinogenic toxicity	CTUh	1.927×10^{-3}	2.447×10^{-3}	3.209×10^{-3}	4.602×10^{-3}	8.328×10^{-3}	8.956×10^{-3}
Respiratory effects	Kg $PM_{2.5}$ eq	1.349×10^{-6}	1.508×10^{-6}	2.041×10^{-6}	2.174×10^{-6}	3.316×10^{-6}	4.655×10^{-6}

Table S16. Environmental and health impact percent changes after substituting the global energy mix with the Swedish energy mix.

Impact Category	Unit	M1	M2	M3	M4	M5	M6
Global warming	kg CO2 eq	14.65958	14.25201	13.12423	10.41731	8.681046	8.28115
Ozone depletion	kg CFC-11 eq	-62.6212	-61.07	-59.7748	-54.5356	-46.8844	-48.1798
Smog	kg O3 eq	-88.5691	-86.1527	-82.1751	-78.4327	-66.7917	-63.2306
Acidification	kg SO2 eq	-89.2461	-88.533	-85.2322	-76.9974	-71.3188	-68.3228

Eutrophication	kg N eq	-84.3569	-82.61	-80.7222	-75.7834	-66.6267	-66.8382
Fossil fuel depletion	MJ surplus	-89.7147	-87.0726	-84.3935	-79.6857	-67.2182	-66.8826
Ecotoxicity	CTUe	-89.6547	-87.1626	-78.376	-79.3543	-67.4169	-54.1687
Carcinogenics	CTUh	-77.8958	-74.1525	-52.6591	-51.5278	-39.9914	-24.0104
Noncarcinogenics	CTUh	-94.2539	-92.8167	-90.7858	-87.2935	-79.1489	-77.9234
Respiratory effects	Kg $PM_{2.5}$ eq	-72.3554	-70.0763	-63.3779	-61.8926	-51.576	-43.1366

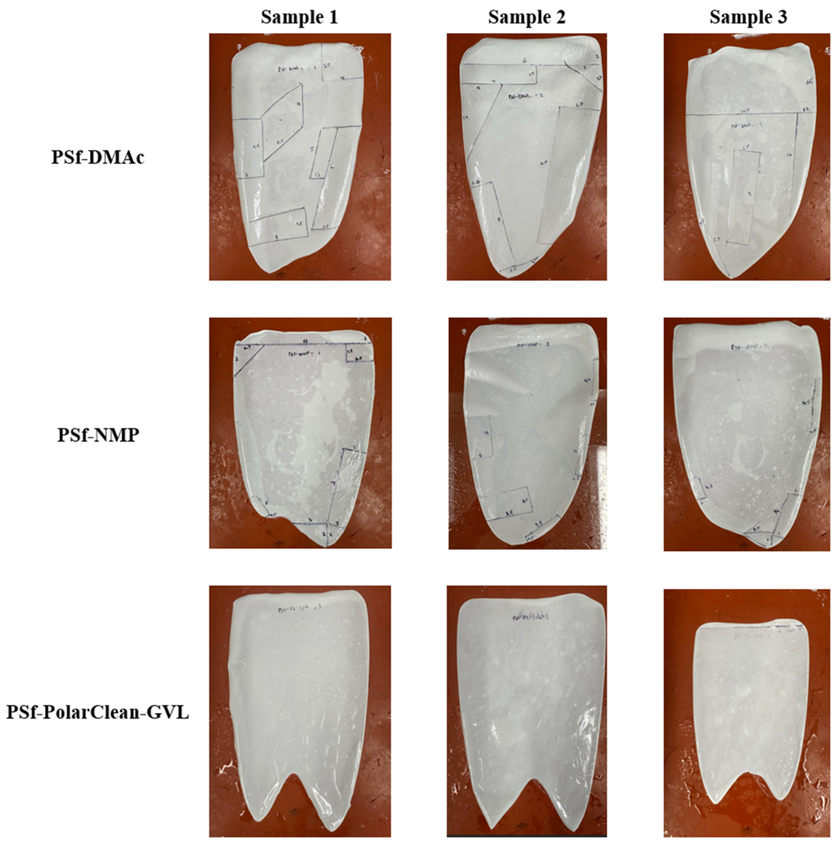

Figure S1. DBE membrane samples prepared using DMAc, NMP, and PolarClean-GVL to estimate material loss due to residual dope solution left on the doctor blade and surface defects (marked on the samples). Material loss calculation results are presented in Table C14.

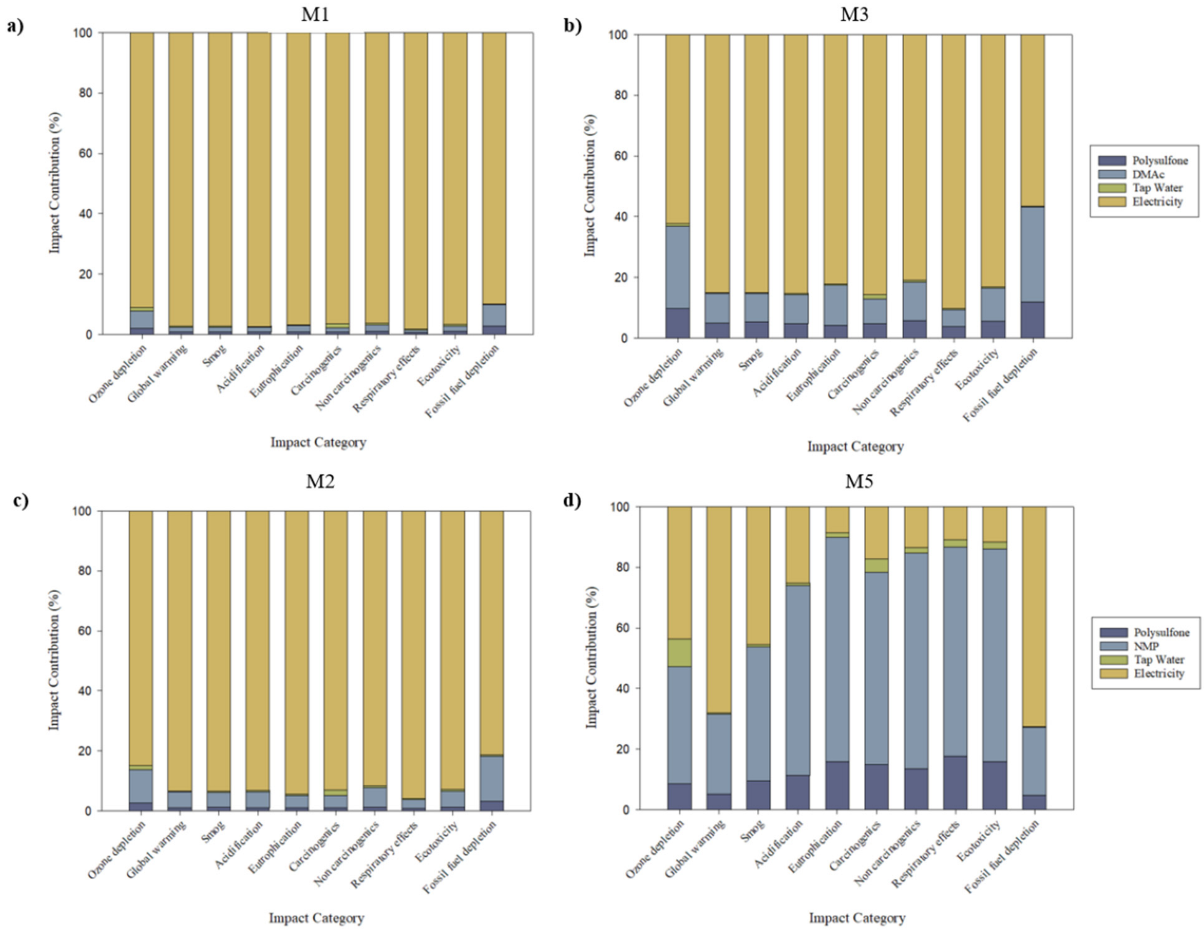

Figure S2. Material impact contributions for the fabrication of 1 m^2 flat sheet of a) M1, b), M3, c) M2, and d) M5.

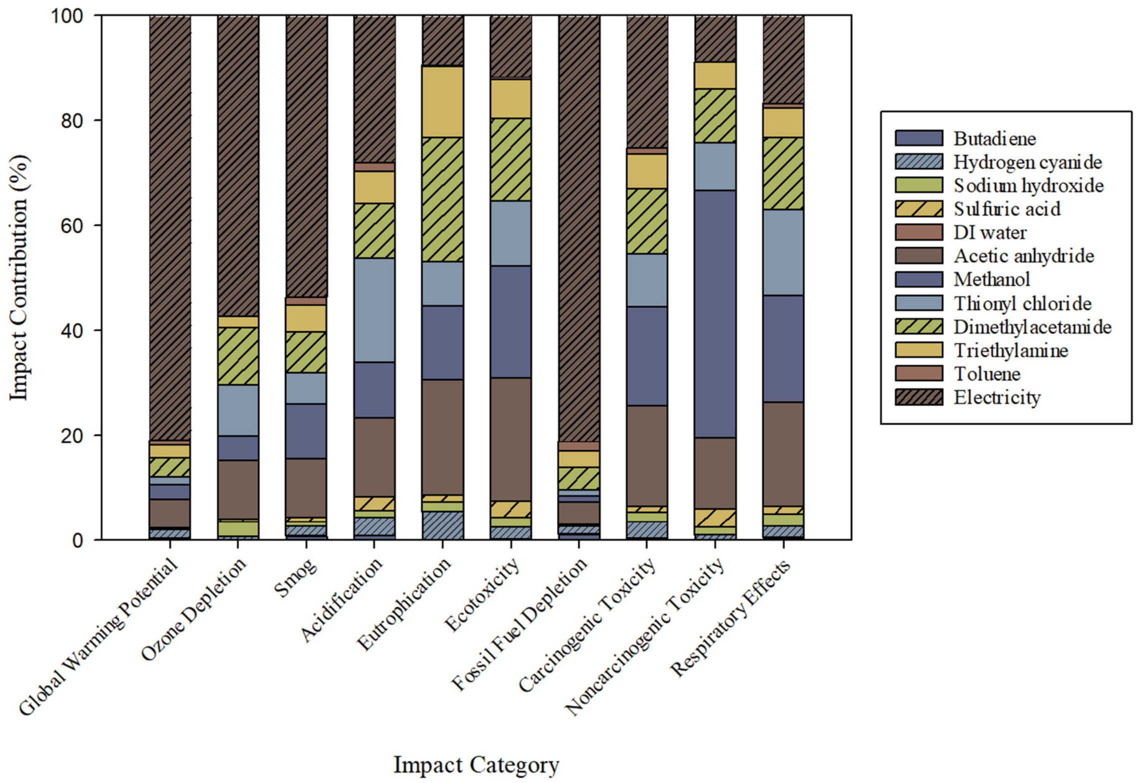

Figure S3. Material impact contributions for the production of 1 kg of PolarClean via PolarClean Commercial Route A1.

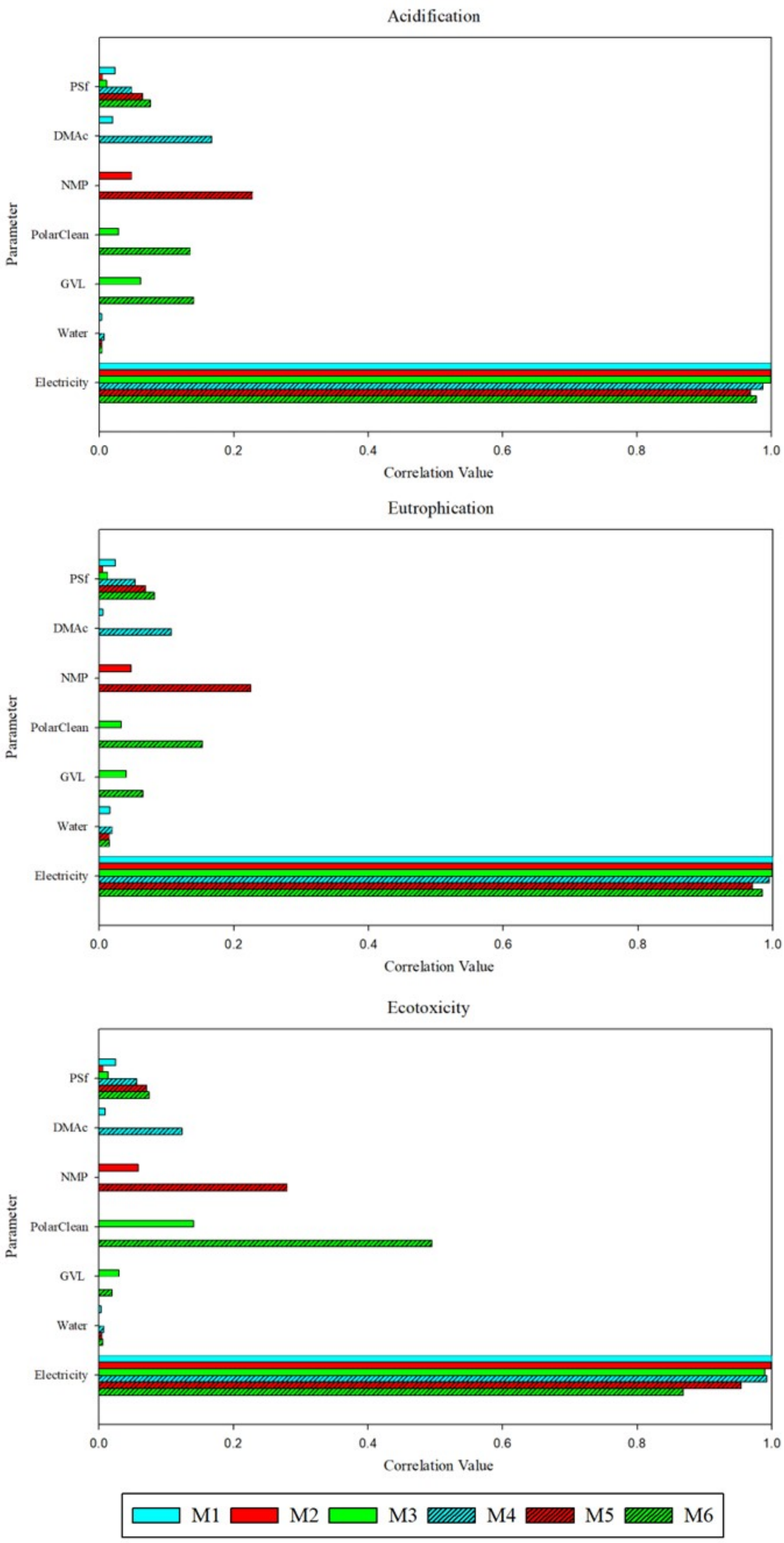

Acidification
Parameter
PSf
DMAc
NMP
PolarClean
GVL
Water
Electricity
0.0
0.2
0.4
0.6
0.8
1.0
Correlation Value
Eutrophication
Parameter
PSf
DMAc
NMP
PolarClean
GVL
Water
Electricity
0.0
0.2
0.4
0.6
0.8
1.0
Correlation Value
Ecotoxicity
Parameter
PSf
DMAc
NMP
PolarClean
GVL
Water
Electricity
0.0
0.2
0.4
0.6
0.8
1.0
Correlation Value
M1
M2
M3
M4
M5
M6

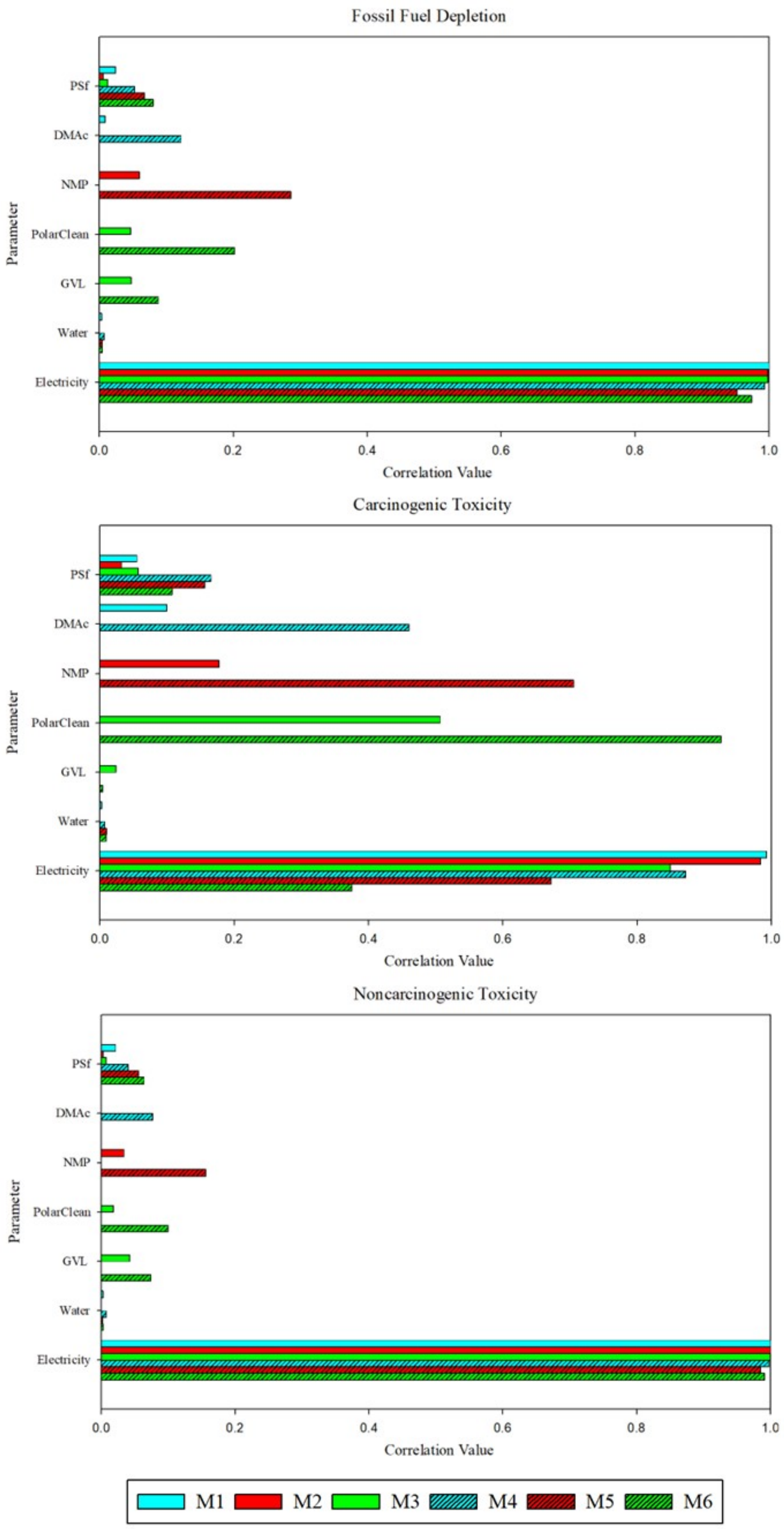
Fossil Fuel Depletion
Parameter
PSf
DMAc
NMP
PolarClean
GVL
Water
Electricity
0.0
0.2
0.4
0.6
0.8
1.0
Correlation Value
Carcinogenic Toxicity
Parameter
PSf
DMAc
NMP
PolarClean
GVL
Water
Electricity
0.0
0.2
0.4
0.6
0.8
1.0
Correlation Value
Noncarcinogenic Toxicity
Parameter
PSf
DMAc
NMP
PolarClean
GVL
Water
Electricity
0.0
0.2
0.4
0.6
0.8
1.0
Correlation Value
M1
M2
M3
M4
M5
M6

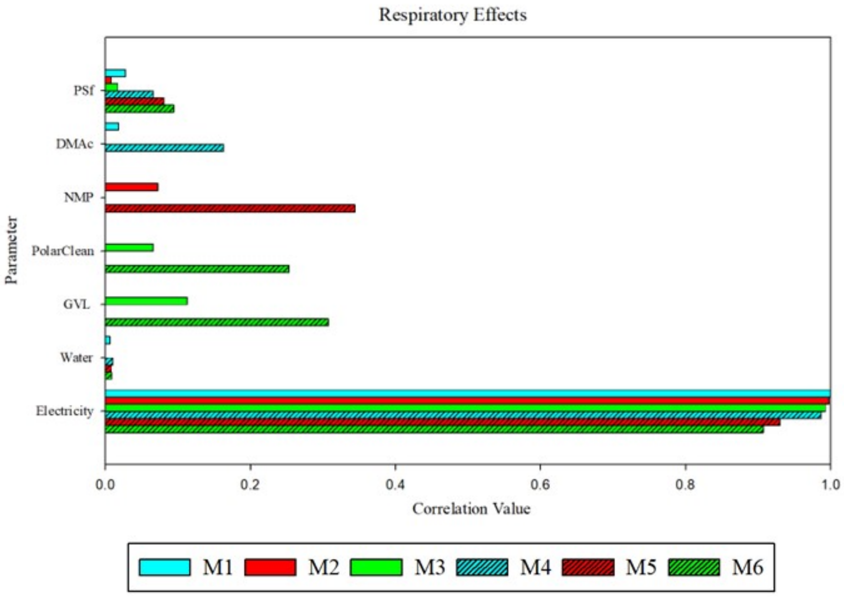

Figure S4. Sensitivity of impacts to membrane fabrication parameters with respect to acidification, eutrophication, ecotoxicity, fossil fuel depletion, carcinogenic toxicity, noncarcinogenic toxicity, and respiratory effects using Spearman's rank coefficient. Note that blue shades denote DMAc, red shades denote NMP, green shades denote PolarClean-GVL, and patterning denotes samples fabricated via SDC. Other sensitivity results are presented in Figure 5.
